# Delay-differential SEIR modeling for improved modelling of infection dynamics

**DOI:** 10.1038/s41598-023-40008-9

**Published:** 2023-08-18

**Authors:** I. N. Kiselev, I. R. Akberdin, F. A. Kolpakov

**Affiliations:** 1 FRC for Information and Computational Technologies, Novosibirsk, Russia; 2 Sirius University of Science and Technology, Sirius, Russia; 3 BIOSOFT.RU, Ltd, Novosibirsk, Russia; 4 Novosibirsk State University, Novosibirsk, Russia

**Keywords:** Computer modelling, Differential equations

## Abstract

SEIR (Susceptible–Exposed–Infected–Recovered) approach is a classic modeling method that is frequently used to study infectious diseases. However, in the vast majority of such models transitions from one population group to another are described using the mass-action law. That causes inability to reproduce observable dynamics of an infection such as the incubation period or progression of the disease's symptoms. In this paper, we propose a new approach to simulate the epidemic dynamics based on a system of differential equations with time delays and instant transitions to approximate durations of transition processes more correctly and make model parameters more clear. The suggested approach can be applied not only to Covid-19 but also to the study of other infectious diseases. We utilized it in the development of the delay-based model of the COVID-19 pandemic in Germany and France. The model takes into account testing of different population groups, symptoms progression from mild to critical, vaccination, duration of protective immunity and new virus strains. The stringency index was used as a generalized characteristic of the non-pharmaceutical government interventions in corresponding countries to contain the virus spread. The parameter identifiability analysis demonstrated that the presented modeling approach enables to significantly reduce the number of parameters and make them more identifiable. Both models are publicly available.

## Introduction

Mathematical modeling of the spread of infectious diseases is a powerful and widely used approach to predict infection, lethality and mortality rates in a certain country or over the world^1^. It may also help reveal what should be the most effective administrative strategies and social containment measures in order to minimize loss of life and productivity and to curb the spread^2–5^. The SIR (Susceptible, Infected and Recovered) method is an often used approach to build epidemiological models^6^. It has been widely applied to simulate the epidemic spread, control mechanisms and impact on economic output of the COVID-19 in different countries^7–10^. SIR-models can be easily extended, for example, to include different aspects of the disease. For instance, in^11^ inclusion of the viral load and the impact on the immune human system into the SIR-model has enabled the identification of potential causes of two-phase exponential growth of the epidemic. Another natural extension is taking into account incubation time of the virus. Such models are usually called SEIR-models where E means exposed^12^.

SEIR-models found enormous application in theoretical studies of diverse aspects of the novel SARS-CoV-2 pandemic. In particular, such models were used to estimate the impact of different lockdown intensities on epidemic spread in China^13,14^, United Kingdom^15^ and Europe (e.g. the Netherlands)^16^ for the year 2020, and even until 2025 for the USA, considering seasonal forcing and cross-immunity from the other betacoronaviruses^17^. The SEIR modeling has also been harnessed to estimate the effect of local and international travel restrictions on the spread of COVID-19 outbreak^18^.

The typical scenario of authorities’ actions (also called NPIs for Non-Pharmaceutical Interventions) simulated in SEIR models is a restriction on the mobility and mass gatherings which reduce the number of contacts in the population and can be represented as an additional multiplier to the infection rate law reflecting social distancing^16^. The numerical value of the control parameter may be changed via discrete events and piecewise functions. Other key NPIs are closing borders and quarantine on entry which diminishes the influx of infected individuals to the simulated region and mass testing for the virus where different modes of testing can be implemented in the model depending on the financial capabilities and government acts and policies (random tests or testing of infected with severe/critical symptoms, or considering contacts of an infected individual etc.). The number of hospital beds and intensive care units (ICU) is another crucial factor in the fight of authority against COVID-19 which should be considered in epidemiological modeling^19^.

Despite the fact that initial results of the numerical study of SEIR models played an essential role in determining both basic laws of the primary development of the COVID-19 pandemic and core characteristics of the current pandemic situation, in the overwhelming majority this type of models use mass action laws to describe the transitions between states (for example, from the incubation period to the symptomatic). Because of that, such models cannot always adequately reproduce the dynamics of such transitions. The methodological constraint of the SEIR models can be solved by using delayed differential equations which are able to explicitly capture the durations of the latent, quarantine, and recovery periods^20,21^. Thus, Shayak and coathours numerically investigated the simplest retarded logistic equation with time delay to model the spread of COVID-19 in a city and demonstrated that solution of the model is significantly sensitive to small changes in the parameter values^22^. At the same time, more conventional SEIR-based delay differential equation models were proposed to reproduce the COVID-19 dynamics in Germany, China, South Korea, India and Japan^23–26^ and to predict the epidemic dynamics in Italy and Spain when it was in its early stages. However, these models did not take into account asymptomatic carriers and non-testing subpopulations as well as the progression of the disease’s severity.

Herein, we propose novel mathematical model based on the model developed by^16^ using differential equations with weighted sums of delayed argument mixed with instant processes, which allow us not only model transition processes adequately to clinically observed data, but also directly quantify the proportion of hospitalized patients with moderate and severe symptoms, on an ICU, asymptomatic, tested and untested among them, which can be compared with the available statistics. The main goal of the study is to present a new approach to model epidemiological processes combining delay-differential terms and instant processes which may be fitted separately from the rest of the model. This approach reduces the number of model parameters and makes them more epidemiologically interpretable and more identifiable compared to classic SEIR approach. The results of numerical analysis and model validation are demonstrated by the example of two European countries, Germany and France.

## Results

### Model structure

**Figure 1:**
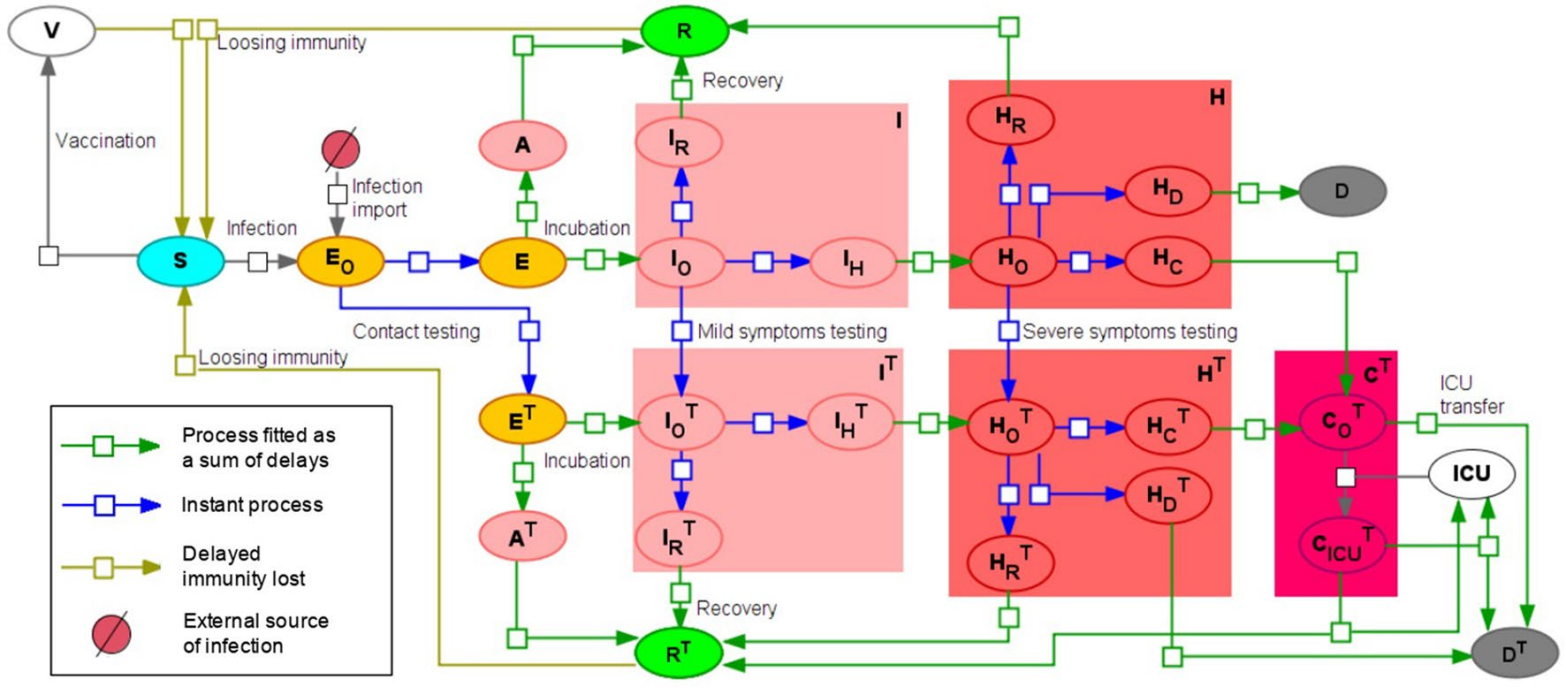
Overall SEIR-like model with instant and delayed processes. All abbreviations of the population groups described in the main text.

The final version of the proposed delay differential equations (DDE) model consists of the following subpopulations or groups (Fig. 1):

1. *S*—susceptible to the SARS-CoV-2 virus.
2. *V*—vaccinated subpopulation, considered to be immune to the virus.
3. *E*—exposed to the virus. After the incubation period they will transit either to asymptomatic or symptomatic. Here we do not use additional subgroups due to equal time intervals for both transitions.
4. A—asymptomatic individuals, which will recover over time but can infect others^27^.
5. *I*—mild symptomatic group. It comprises three subgroups: with onset symptoms ($${I}_{O})$$ then they are instantly divided into those who will recover ($${I}_{R})$$ and those who will progress to the severe symptomatic ($${I}_{H})$$. Transition is done according to the fraction of severe symptomatic among those who show any symptoms ($${F}_{H})$$.
6. *H*—severe symptomatic group which comprises four subgroups: 1) with just onset symptoms ($${H}_{O}$$). They instantaneously transit into subgroups of individuals who will eventually recover ($${H}_{R})$$, die ($${H}_{D})$$ or progress to critically ill ($${H}_{C})$$. Transitions are performed according to the disease lethality ($${F}_{D}$$) and the fraction of critically ill ($${F}_{C}$$).
7. *C*—critically ill group where ICU is required in order to recover. If no ICU is available these patients will die. All critically ill patients are considered to be automatically tested for the virus infection.
8. *R*—recovered from COVID-19.
9. *D*—deceased due to COVID-19.
10. All infected subgroups (except critically ill) also have “registered” or “tested for COVID-19” counterparts: ${A}^{T}, {E}^{T},{I}^{T}, {H}^{T},{R}^{T},{D}^{T}.$ In the model patients may be tested at three different stages (1) when being exposed to the virus. It is done through contact tracing procedures. Percentage of exposed to the virus who will be tested and registered is set by ${T}_{E}$ parameter; (2) upon symptoms onset. Percentage of mildly symptomatic individuals who will be registered is given by parameter ${T}_{I}$; (3) upon severe symptoms onset. Percentage of severely symptomatic individuals who will be registered is given by parameter ${T}_{H}$.

Most transitions in the model are described as either instant processes or as preliminary fitted processes (blue and green arrows, correspondingly, in Fig. 1). To fit delayed processes we used data from^28^ for incubation period and^29^ for other epidemiological processes.

**Figure 2:**
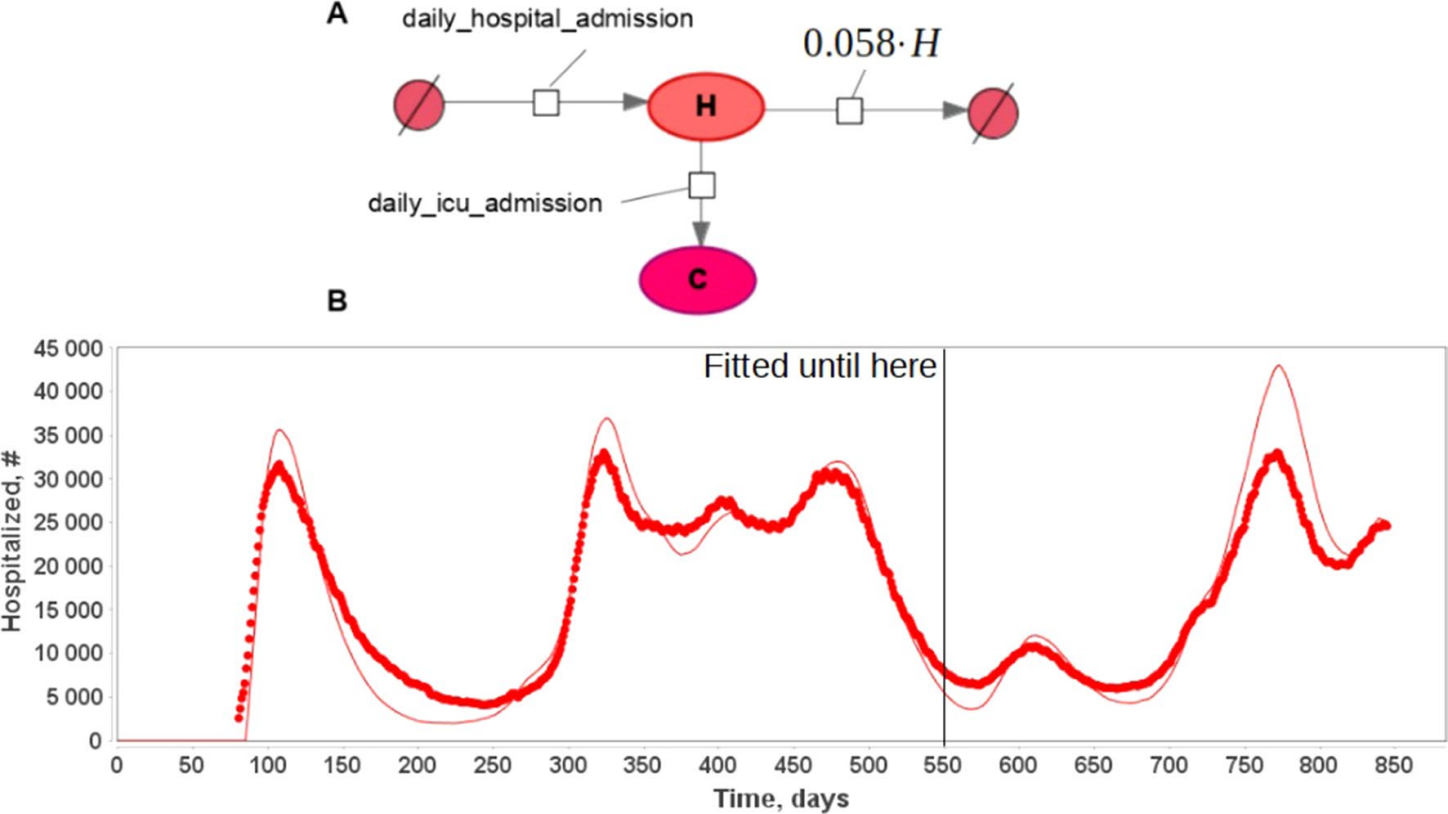
(**A**): Partial model of the hospitalization due to Covid-19 using delay equations for hospital release. Data on daily hospital and ICU admission taken from ourworldindata.org. (**B**): Results of the model fitting to the number of hospitalized patients in France. Model was fitted until time = 550 days.

Process of release from a hospital may be fitted using data provided by Our World in Data for France for recovery\dying in hospitals. To this end we constructed a partial model describing the process of admitting hospital, transition to ICU and leaving hospital (Fig. 2). Given daily numbers of the hospital admission, daily number of ICU admissions and daily number of hospital patients we fitted the process of leaving hospital utilizing formula (3) (see “Methods”). It should be noted that we assume that all severely ill patients are tested and moved to the hospital. However, it is not always the case and should be addressed in the updated version of the model.

Overall scheme of the hospitalization model in SBGN format as well as a result of the model fitting to statistical data on hospitalization in France are presented in Fig. 2. It should be noted that hospital stay duration is shown to be the same for the whole pandemic duration and not dependent on the virus strain.

There are also four transitions in the model treated differently:

1. *Duration of the protective immunity.* We imply that the immunity acquired either after recovery or vaccination lasts 180 days according to the average experimental evaluations^30,31^.
2. *Vaccination*. Based on the known statistical data we established the number of individuals vaccinated each day in a certain country. For model purposes we allow the vaccination only for susceptible individuals (either never infected before or those who lost their immunity through time). The kinetic law for this process is zeroth-order: $dV/dt = {k}_{V}$ with ${k}_{V}$ changing each day based on the tabular data for the certain country. We also assume that vaccines have 100% efficiency immediately after the first dose, and the immunity via vaccination declines according to the duration period (see item 1).
3. *ICU admittance*. We considered this process to require a free ICU and be instant in most cases. However, lower value of the kinetic constant may be used to reflect the fact that not everyone who needs ICU gets it:
  $$d{C}_{ICU}/dt={k}_{ICU}\cdot ICU\cdot C, {k}_{ICU}\le {K}_{Large}.$$
4. *The infection process*. Transition from susceptible to exposed is defined using Total Infection Coefficient (*TIC*) which we calculated similar to the model described in^16^ with modifications:
  $$dE/dt = TIC\cdot S \cdot \frac{1}{SIF},$$
  $$TIC=\frac{CF\cdot IC}{P}\cdot {\sum }_{j\in \{A, E, I, H\}}(j+{{Q}_{j}\cdot j}^{T}),$$
  where $CF$—average number of contacts for individuals per day in the simulated region which can be obtained from statistical surveys, $IC$—a probability to be infected upon contact, ${Q}_{j}$—quarantine coefficient for corresponding group. Only registered individuals are subject to quarantine, $SIF$ is a stringency index factor calculated based on the Stringency Index^32^ which reflects government NPIs and imposed limits such as mask regime, limit on mass gatherings, school closing etc. Stringency index ranges from 0 (no interventions) to 100 (maximum possible interventions). In the current study we recalculated it to SIF as follows:
  $$SIF=100/(100-{SI}_{Effect}\cdot SI(t-{SI}_{delay})),$$
  where ${SI}_{Effect}\in [\mathrm{0,1}]$ is a parameter describing the efficacy of employed NPIs implying different adherence to the NPIs during the COVID-19 pandemic as well, ${SI}_{delay}$—time delay between government interventions enacting and their effect. See more details about Stringency Index in the Methods section.
  The developed models may be accessed and simulated at https://gitlab.sirius-web.org/covid-19/dde-epidemiology-model via user interface or Jupyter Notebook.

### Simulation results

The final version of the DDE based model was fitted to COVID-19 epidemic data in Germany and France from 01.01.2020 (model time t = 0) to 31.12.2021 (model time t = 730). All model parameter values are presented in Supplementary material 1 (Tables S1–S6). We have divided the overall time duration into four intervals or waves. Values of some model parameters were changed between waves to reflect changes in the pandemic progression. The most significant changes were made to the infection coefficient which causes a spike in new cases and reflects the spread of new more contagious SARS-Cov-2 variants^33–36^.

1. The First wave: This interval starts somewhere in January 2020. From this time point infected individuals started to arrive in the country in significant amounts. Patient zero in Germany entered the country on 20 January and was registered on 27 January^37^. We assumed in the model that import of infection to Germany began on 20 January. This import was ended on 16 March 2020 (t = 76), when the European Union as a whole announced the closure of all its external borders to non-citizens^38^. We assumed the import rate to be linearly increasing during that time period. Maximum number of infected individuals per day was estimated to be 500 individuals per day just before borders were closed.

For France the first case was identified on 24th January. However, individuals infected by SARS-CoV-2 were present as early as December 2019 according to some sources^39,40^. Unfortunately, we do not have data on how many infected individuals arrived in France or Germany before borders were closed on 16 March 2020. We kept the same number of persons per day for France as for Germany and estimated the start of infection import to France to be 15 January.

2. The second wave—starting from summer 2020 the number of new cases began to rise again implying the second epidemic wave in the region with many more registered cases. It may be attributed to a new European strain (EU1) emerging in both countries. However, its transmissibility is considered to be the same as for the original variant^41^. Another reason is relaxing anti epidemic restrictions which can be traced by lower levels of the Stringency Index. In both models it caused a second wave in accordance with existing statistics. Despite the fact that the number of cases is much higher than during the first wave, the number of hospitalized patients is roughly the same. In both models it was reflected by changing the fraction of severely ill among symptomatic patients ${H}_{F}$. New value was fitted according to the statistical data for patients in hospitals and ICU. New values were set in the model at time = 200 (20.07.2020) which agrees well with the appearance of the new strain in both countries according to the covariants.org web site. However, it should also be noted that the number of cases at the first pandemic wave could be dramatically underestimated due to limitations on testing compared to further deployment of the testing system. To estimate the correct number of initial cases data on seroprevalence during the first wave is required which is also limited.

3. The third wave—starting from early 2021 a new significant rise in numbers of cases begins in both countries. It may be connected with the spread of new strains of the virus. Indeed, new virus lineage with additional mutations in the spike region, B.1.1.7 strain^42^, was rapidly spreading in some European countries at this time period. This Alpha strain is much more contagious and has increased mortality rate according to the cohort study^33–35^. This was modeled by multiplying all probabilities to be infected upon contact by the same multiplier. The multiplier’s value was implied to be the same for both countries and fitted to be 1.6. Thus, the probability of being infected upon contact is 60% larger for the new variant which is consistent with the estimated range of the transmissibility of the Alpha strain compared to the predecessor lineage^43^. Fraction of severely ill ${H}_{F}$ was not changed at that time, as the previous value still agreed well with statistical data. Start of the third wave in the model was fitted for both countries on two different dates. Here it should be noted that the obtained profile of new cases for France does not agree well with the statistical data from ourworldindata.org.

4. The fourth wave starts in June 2021 and can be attributed to another B.1.617.2 virus variant (Delta) which is significantly more contagious than previous ones. New infection coefficient was fitted to be 2.3 times larger than for the Wuhan strain which agrees with estimates in the published data^44,45^. Fraction of severity ill ${H}_{F}$ was fitted to be even less than for two previous strains to reflect a lowered ratio of hospitalized patients to registered. Despite the risk of hospital admission for COVID-19 was approximately doubled in patients with the Delta compared to the Alpha strain^36^, the overall hospital admissions involving COVID-19 in 2021 were significantly lower than in 2020 (e.g., see ONS data on COVID-19 latest insights: Hospitals. 9 June 2022). Apparently, the increased number of vaccinated people, protective effectiveness of the developed vaccines in preventing SARS-CoV-2 infections as well as a much greater proportion of the population cohort who is recovered from COVID-19 by the moment of Delta variant’s emergence in 2021 ensured or can explain the ratio decline in this year compared to the first pandemic year. Once again, the time point at which new parameter values were introduced into the model was fitted for both countries for two different dates to obtain the required profiles of new cases.

**Figure 3:**
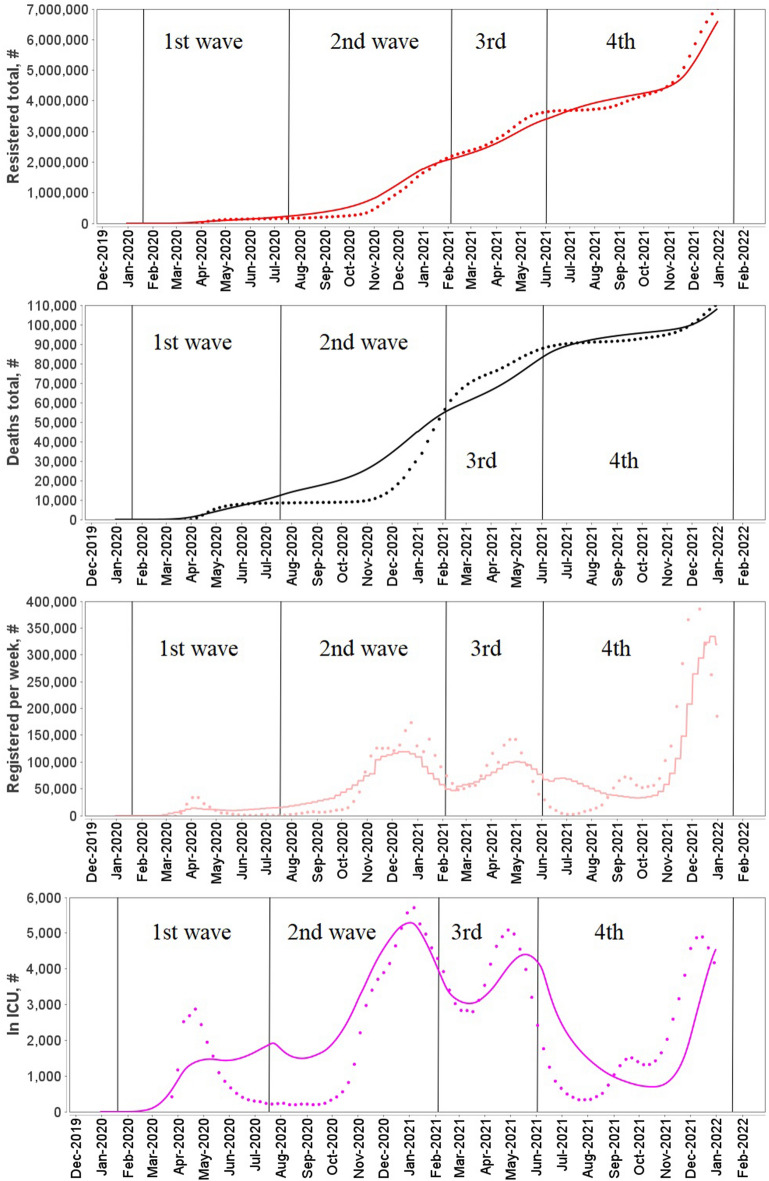
Simulation results and statistics from ourworldindata.org for Germany for 2020–2021 years.

**Figure 4:**
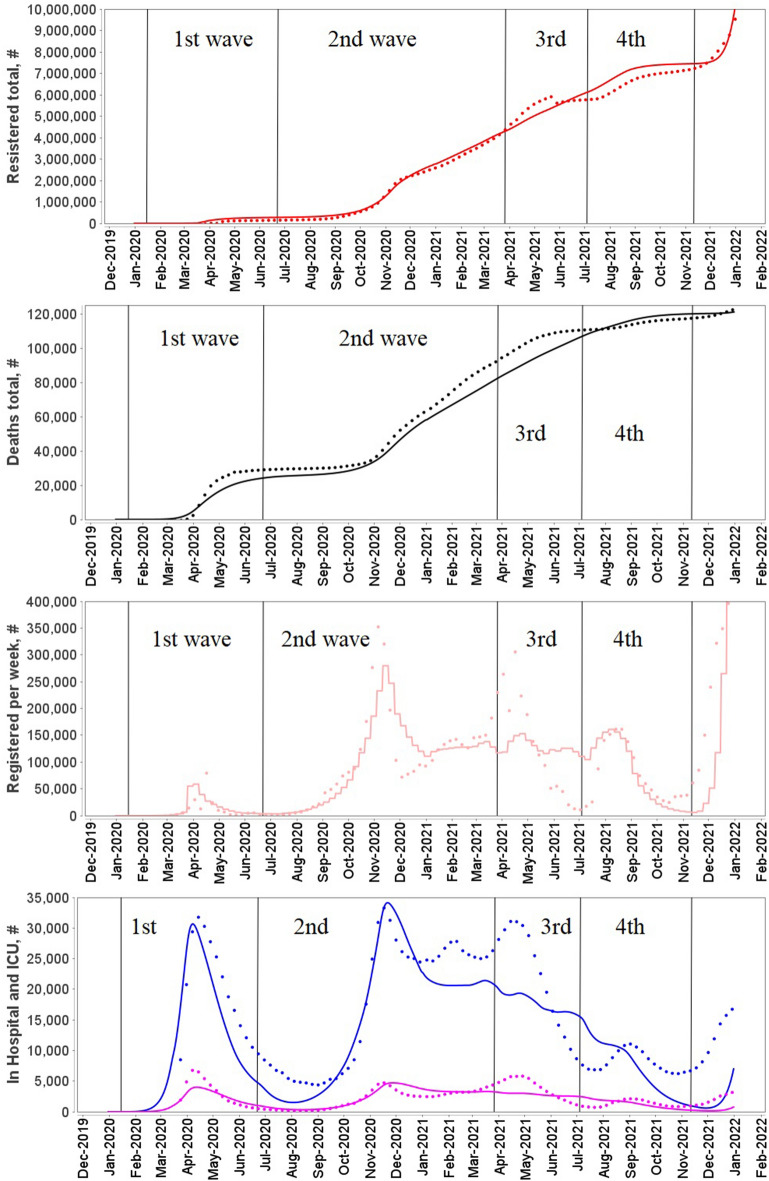
Simulation results and statistics from ourworldindata.org for France for 2020–2021 years.

Described four waves cover the first two years of the pandemic. As can be seen from simulation results (Figs. 3 and 4) the model accurately reproduces the reported new cases per week and total number of cases as well as the number of hospitalized patients on ICU and total deaths in each country over time of the pandemic for two years of the pandemic.

Sources used for deriving model parameters include: data on average number of contacts between individuals^46^, fatality rate on ICU^47,48^, transimissibility of the original virus strain^49–51^. Particular values and ranges of parameters may be found in Supplementary material 1.

### Automatic model generation for other countries

Consequent development of our approach is automatic generation of epidemiological models for other countries (both European and non-European). Most of the model processes are fixed by fitting weighted sums of delays (see Methods). Because of that, the number of parameters whose values should be estimated is significantly lowered. That, in turn, allowed us to carry out estimation procedures automatically for other countries using statistical data provided by Our World in Data. It is worth to note that generated models are preliminary and further fine-tuning procedure is required to quantitatively reproduce observed epidemiological dynamics. However, it provides the fast generation of the initial version of epidemiological model for a certain country.

Automatic model generation goes as follows:

1. Copy of the base model is created.
2. Tabular data for a given country is derived from https://covid.ourworldindata.org/data/owid-covid-data.csv.
3. It is adapted to given country with following parameters:
  - Size of the population.
  - Number of vaccinated people each day.
  - Date when first case was registered ${T}_{0}$.
  - Start of infection import is set to ${T}_{0}-15$ days.
  - Cancellation of the infection import (date when sufficient restrictions on borders were imposed) is set to ${T}_{0}+30$ days.
  - Date when testing was started is set to 35 days (i.e. 4th February).
4. Parameters of the generated model are estimated so that weekly new cases simulated by the model agree with statistical data for a particular country. At the current stage, we estimated the model parameters at the first year of the epidemic in a certain country. Parameters, which values were estimated, are presented in Supplementary material 2 (Table S1).

Simulation results of the automatically generated and fitted models for 12 countries are presented in Supplementary material 2 (Figures S1–S4). It is worth emphasizing that simulation results quite well agree with statistical data for most European countries, while optimization of the model describing COVID-19 epidemiology in Non-European countries like Brazil and Argentina was not able to adequately reproduce the observed trajectory of the epidemics. For example, one can see that the number of total cases in South Korea agrees well with real data, but the model failed to demonstrate the first two waves of pandemic. That is due to the fact that those waves are short and presented only by a few data points (2–3 weeks) and automatic parameter estimation ignores these small waves. Probably that manual model fitting and optimization are required in those cases. Of course, the generated models are not a final in silico tool to describe and predict epidemics even in European countries, but they may serve as a base for models of COVID-19 epidemic in corresponding countries.

## Discussion

We have proposed the methodology to overcome some shortcomings of the classic SEIR-based epidemiological models via the novel epidemiological model which utilizes DDEs to take into account different time scales of epidemiological processes and instantaneous splitting procedure to describe competing processes. The essential benefits of the developed model are:

1. Most of the epidemiological processes (symptoms onset, recovery, dying etc.) are described using kinetic laws with delayed arguments. These modeling processes can be fitted separately from the rest of the model and applied kinetic laws provide more precise reproduction of real properties of those processes than mass-action laws with a single parameter.
2. If a model has two or more competing transitions, a division into separate subpopulations removes their undesired mutual influence and allows to simulate fast and slow transitions with correct fractions of patients undergoing each transition.
3. Model parameters that are not parts of fitted processes described previously have direct mechanistic meaning (i.e. disease lethality, susceptibility, probability of different symptoms severity) and can be drawn from the statistics.
4. Overall decreasing of the parameters number makes them more identifiable as demonstrated in comparison between delay-based and classic models in this study. It also allows for more fast and simple adaptation of the model to other regions and countries.

Combination of these advantages makes the model more reliable with real properties of the pandemic and eliminates most of the abstract parameters usually used in SEIR-like models. It is worth noting that there are other studies addressing these issues using Erlang distribution^52^ and delayed equations^53^. However, to our best knowledge, there are no publications demonstrating DDE-based approaches with weighted components of delayed arguments and instant transitions. Thus, we believe the proposed COVID-19 model and simulation results may be of interest to both experts in epidemiological modeling and a more general audience.

The final version of the DDE-based model describes many aspects of COVID-19 pandemic such as vaccination, loss of immunity over time, asymptomatic carriers, testing for virus, hospitalization for severely ill and intensive care required for critically ill. The model of course still has a number of abstract parameters reflecting government NPIs, conducted testing procedure, imposing quarantine for those who were detected as infected with the virus and importing infected individuals into the modeling region or country. Particularly, parameters describing fractions of different symptoms’ severity should be correctly attributed to the patient's age that requires extension of the model.

One of the crucial issues in the case of COVID-19 epidemiological modeling is to correctly transfer government NPIs (limit on mass gathering, lockdown, curfew, etc.) into the model parameters. One can easily see that introduction of a “social distance” multiplier to the infection rate and fitting its value to experimental data enables it to reproduce almost any observable epidemiological trajectory. In order to tackle this problem we tried to utilize the Stringency Index^32^ instead of trying to fit the “social distance” factor over time of the pandemic in a certain country. However, we still have quite abstract aggregated numerical values of the indicator. Thus, further step for the model development in this direction is to use individual components or NPIs of the stringency index and attempt to assess their individual effect on the epidemic dynamics.

It should be noted that the main focus of the study is to present a novel, as far as we know, combination of delay-differential terms and instant processes which may be fitted separately from the rest of the SEIR model in order to simulate the pandemic. However, this approach and developed model for COVID-19 epidemic have some constraints and limitations. Firstly, the delay-differential version of the SEIR model like others assumes population homogeneity and it could be overcomed only by application of agent-based modeling approach. Secondly, Covid-19 pandemic shows wave-like behavior in any country and the model analysis and fitting show that predictive power of this type of SEIR model like others is low and COVID-19 trajectory can not be predicted by the pre-fitted model without additional modifications taking into account an emergence of new viral strains with different epidemiological characteristics and consequent refitting of the model. So we associate each wave with appearance and spread of new more contagious variants of the virus and model it by changing infection parameters. However, it may be modeled more correctly using a modified version of the model with two or more similar modules where each of them is containing a full disease progressing scheme taking into account separate strains. This is also a part of our roadmap in the development of the model. The model fitting to statistical data for both countries demonstrated the decrease of the infection fatality rate during the COVID-19 epidemic which corresponds to early statements that the fatality rate of the Delta or B.1.617.2 variant of COVID-19, for example, is lower than the original variant. However, it might be caused by the age of unvaccinated people who were infected by the Delta virus strains and hospitalized with severe symptoms. According to the report published by Public Health England^54^, for instance, the majority of COVID-19 cases caused by the Delta variant were detected in people under 50 years old in the UK which are less likely to die from COVID-19 compared to those older than 50. So the current comparison of the case fatality rate of the B.1.617.2 variant with that of the wild-type virus is biased due to vaccination strategy in some European countries and age stratification of the population. This statistical misleading indicates the necessity to specify the developed model for each age group and integrate them in a more complex DDE model considering age distribution in a certain country. In addition to that, the vaccination scheme is oversimplified in the current version of the model and does not consider essential factors like two-stage and booster vaccinations, the vaccine efficiency against different strains (in regards to protective immunity to be infected and to show more serious symptoms), vaccination of asymptomatic cohort, different strategies or vaccine campaigns specific for particular country. So the roadmap for the extension and further model development includes the next biological aspects of the virus and epidemiology of the COVID-19 pandemic:

- Age-specific modules (infection and death rates, hospitalization and severity of the disease, B and T cell response) according to the statistical data^55^.
- Explicit strain emergence with other viral indicators like infectivity, resistance to neutralization, vaccine effectiveness in the population^36,56–62^.
- Waning B and T cell immunity and neutralization activity of specific antibodies^63–66^.
- Superspreading events^67^.
- Different vaccination strategies and its effectiveness against infection and severed outcomes for emerging viral strains.

## Methods

### SEIR-like model

The overwhelming majority of SEIR-like models uses the mass action kinetic law (mainly, the first-order rate law) for transitions between different stages of a disease(e.g. between exposed and infectious periods). Explicit drawbacks of this approach are that:

1. Parameters of those reactions are quite abstract and can not be easily related to real biological characteristics of the virus.
2. The model fails to correctly represent processes delayed in time.

**Figure 5:**
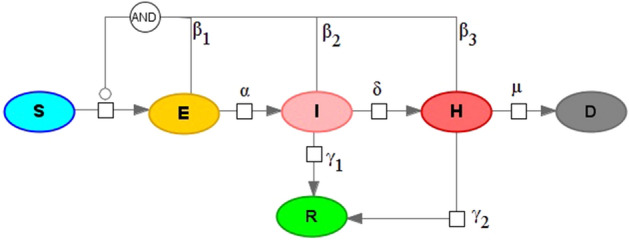
SEIRHD model with mass-action kinetics.

Here we will try to address those two problems. In the current study we use SBGN—Systems Biology Graphical Notation^68^ for visual representation of mathematical models. Let's consider a SEIR-like model with two levels of symptom severity (Fig. 5).

Model equations:

1 $$\left\{\begin{aligned}\frac{dS}{dt}&=-\frac{\left({\beta }_{1}\cdot E+{\beta }_{2}\cdot I+{\beta }_{3}\cdot H\right)}{\left(S+E+I+R+H+D\right)}\cdot S,\\ \frac{dE}{dt}&=\frac{({\beta }_{1}\cdot E+{\beta }_{2}\cdot I+{\beta }_{3}\cdot H)}{(S+E+I+R+H+D)}\cdot S-\alpha \cdot E,\\ \frac{dI}{dt}&=\alpha \cdot E-{\gamma }_{1}\cdot I-\delta \cdot I, \\ \frac{dR}{dt}&={\gamma }_{1}\cdot I+{\gamma }_{2}\cdot H, \\ \frac{dH}{dt}&=\delta \cdot I-\mu \cdot H-{\gamma }_{2}\cdot H, \\ \frac{dD}{dt}&=\mu \cdot H.\end{aligned}\right.$$

With initial values: $S(0)={S}_{0} , E(0) ={E}_{0}, I(0)=R(0) = H(0) = D(0) = 0,$

where S—susceptible population, E—exposed (in incubation period) population, I—infected (with mild symptoms) population, H—infected (with severe symptoms), R—recovered population, D—dead individuals, ${\beta }_{1},{\beta }_{2},{\beta }_{3}$—infection rates for different contagious groups, a—symptom onset rate, $\delta$—symptoms worsening rate, $\mu$—death rate, ${\gamma }_{1},{\gamma }_{2}$—recovery rate (for mild and severe symptoms respectively).

### Duration of processes

A numerical value of the parameter $\alpha$ in the model (1) is related to the median incubation period in the population. For example, if we set $\mathrm{\alpha }=\mathrm{ln}\left(2\right)/5.1$ then 50% of individuals who were exposed to the virus at time t = 0 will have become symptomatic at time t = 5.1 days.

2 $$\frac{dI}{dt}=-\frac{dE}{dt}=\frac{\mathrm{ln}\left(2\right)}{5.1}.$$

**Figure 6:**
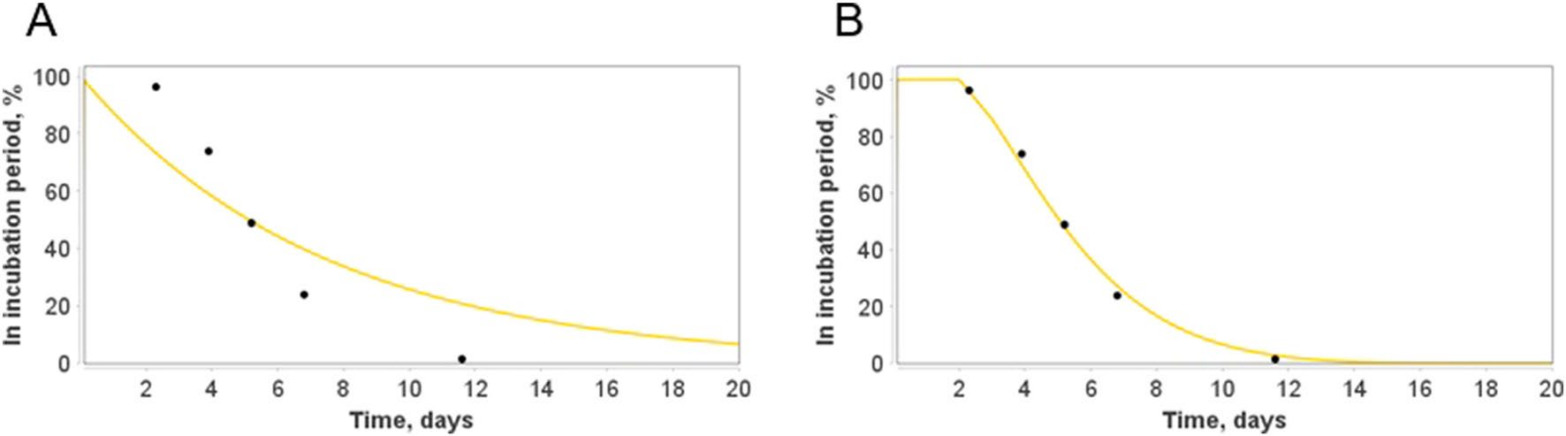
A comparison between two models of the incubation period: (**A**): based on the mass-action kinetics law (2). (**B**): using weighted sum of delays (4). Statistical data of the incubation period quantiles are taken from^28^.

Distribution of incubation period in this model compared to the experimental data from^28^ is presented in Fig. 6A. One can easily observe that it is inconsistent with statistical data on incubation period. For example, according to the first-order model, almost 10% of the infected have their symptom onset within one day of the infection which does not match the data..

Unfortunately, having only one parameter $\alpha$ we can not fit the curve to this statistical data. This is also the case for other processes delayed in time with certain distribution of length.

A possible solution to overcome the issue is use of different forms of the kinetic law:

3 $$ \frac{dI}{{dt}} = - \frac{dE}{{dt}} = \mathop \sum \limits_{i = 0}^{m} k_{i} E\left( {t - \vartriangle_{i} } \right). $$

Herein, we use a weighted sum of the delayed number of exposed individuals. We have a 2*m parameters which can be estimated to reproduce an experimental data. Typically, m = 1 or m = 2 is enough to comprehensively fit the data, keeping the number of parameters reasonably low.

For example, to fit the data from^28^ we employ only two delays:

4 $$\frac{dI}{dt}=-\frac{dE}{dt}=0.14E\left(t-2\right)+0.42E\left(t-3\right).$$

Simulation results of the incubation period’s model demonstrating differences between theoretical curves obtained using two methodologies are presented in Fig. 6.

Another benefit of the time-delay based approach is an opportunity to reproduce an experimental data on diverse epidemiological processes (incubation period, recovery, worsening of symptoms from mild to severe and others) once and separately from the rest of the model structure based on the known distributions of duration of those processes for particular infectious disease only.

### Competing processes

**Figure 7:**
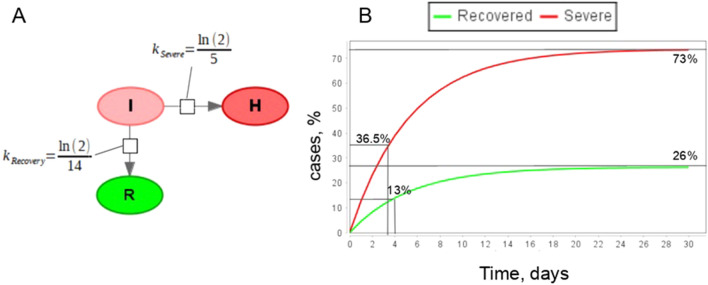
The simple mass-action model with two competing processes. (**A**): SBGN representation of the model, here I—infected individuals, R—recovered, H—patients with severe symptoms; (**B**): Simulation results of the model.

Another opportunity to improve the original model and bring it closer to reality is to consider different possible transitions from the same subgroup. For example, infected patients may either recover or progress to severe symptoms (Fig. 7). In that case parameters $\delta$ and ${\gamma }_{1}$ are related not only to durations of corresponding processes but to recovery rate for mild symptomatic (or fraction of severe symptomatic among mild symptomatic). An issue with two alternatives arises when the fast process has less probability and therefore smaller fraction of patients involved in this direction of the infectious process. According to the statistics^29^, the process of worsening of symptoms (median time is 5 days) is faster than recovery (median time equals to 14 days) implying that $\delta$ value should be larger than ${\gamma }_{1}$ value. As in the previous subsection, we may set these parameters as $\delta =ln(2)/5, {\gamma }_{1}=ln(2)/14.$ However, in that case (Fig. 7) the model will show that the fraction of patients who transit to the severe symptoms is larger than the fraction recovered which is not the case in reality^69^.

**Figure 8:**
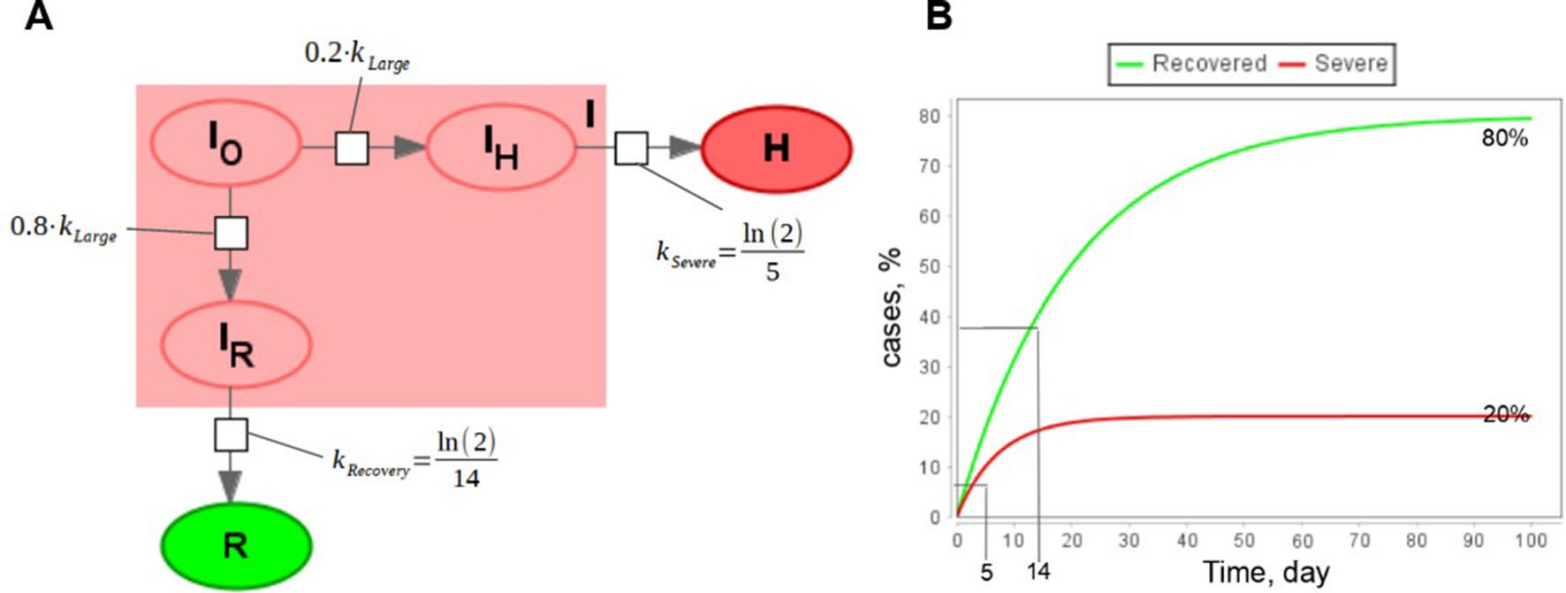
A part of the epidemiological model with alternative competing processes. (**A**): Visual representation in SBGN format; (**B**): Simulation results of the model for fractions of recovered and patients with severe symptoms.

The possible solution to overcome the discrepancy is to consider those two processes separately. Firstly, patients will instantaneously transit from “Symptomatic” to “Symptomatic who will recover in future” ($${I}_{R})$$ and then from $$I$$ to R. In similar way another fraction of symptomatic patient will instantaneously transit to “Symptomatic who will need hospitalization” ($${I}_{H})$$ and only afterwards from $$I$$ to $$H.$$ The updated model is presented in Fig. 8 and corresponding model equations are:

5 $$\left\{\begin{array}{l}\frac{d{I}_{O}}{dt}=-{k}_{Large}\cdot {I}_{O},\\ \frac{d{I}_{R}}{dt}={k}_{Large}\cdot {F}_{R}\cdot {I}_{O},\\ \frac{d{I}_{H}}{dt}={k}_{Large}\cdot {F}_{H}\cdot {I}_{O}.\end{array}\right.$$

where ${F}_{R}=0.2$—a fraction of infectious individuals who will not have worse symptoms. ${F}_{H}=0.8$—a fraction of infectious individuals who will have worse symptoms. ${F}_{R}+{F}_{H}=1.$ ${K}_{Large}$—constant value which is large enough to render reactions instant.

The total number of individuals with mild symptoms is calculated as a sum of all subgroups:

$$I ={I}_{O}+{I}_{R}+{I}_{H}$$

**Figure 9:**
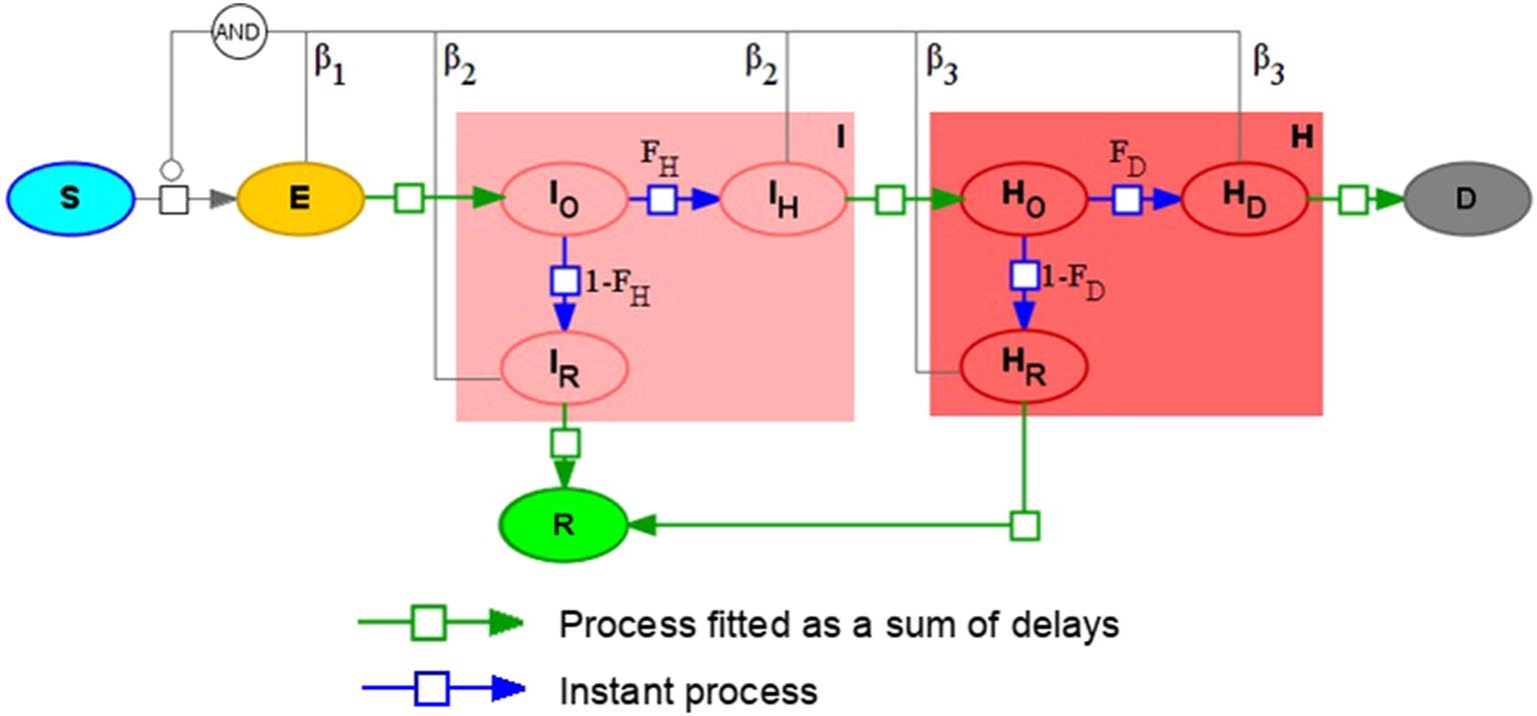
SEIR-like model with instant and delay-based processes (2).

This technique can be combined with delayed equations described in the previous section. Thus, we can construct a version of the model (1) taking into account the duration of some infectious processes and the existence of competing processes. The final version of the model is presented in Fig. 9.

Advantage of the updated model is that instead of 8 parameters which do not explicitly correspond to real characteristics of infectious processes and has to be fitted to experimental data we have only three parameters, two fraction parameters: ${F}_{H}$—fraction of symptomatic individuals with severe symptoms, ${F}_{D}$—disease lethality that can be drawn from statistical data and processes that are fitted to experimental data separately from the rest of the model. Comparison is given in Table 1.

**Table 1:** Comparison of parameters in classic SEIRHD model and delay-based model.

Modeled process	Parameters	
	SEIRHD model (1)	Delay-based model (2)
Exposure to the virus	Infection rates β_1_, β_2_, β_3_	Infection rates β_1_, β_2_, β_3_
Incubation period	α	Fitted process
Recovery with mild symptoms	γ_1_	Fraction of severe symptomatic F_H_, fitted process
Progress to severe symptoms	δ	Fraction of severe symptomatic F_H_, fitted process
Recovery with severe symptoms	γ_2_	Disease lethality F_D_, fitted process
Death	μ	Disease lethality F_D_, fitted process

### Comparison of the model parameters

Advantage of the updated model is that only three epidemiologically interpretable parameters—infection rates ${\beta }_{1}, { \beta }_{2}, {\beta }_{3}$ have to be fitted to experimental data instead of 8 parameters $\alpha ,{ \beta }_{1} ,{\beta }_{2} ,{\beta }_{3}{ ,\gamma }_{1} ,{\gamma }_{2} ,\delta ,\mu$ which do not explicitly correspond to real characteristics of infectious processes. We also have two fraction parameters which values may be explicitly derived from statistics: ${F}_{H}$—fraction of symptomatic individuals with severe symptoms, ${F}_{D}$—disease lethality. All other parameters of the new model are fitted preliminary to experimental data on corresponding processes separately from the rest of the model. Comparison is given in Table 1. Decreasing of parameters number also leads to better identifiability of remaining parameters. In order to demonstrate that we fitted two type of the SEIR model (classic and delay-differential) to data on the first 180 days of the pandemic in Germany in 2020. We simulate a start of the pandemic by the simplistic way and use two additional parameters: $Start$—day, when the infection was imported to the country and ${E}_{Start}$—a number of individuals in incubation period imported to the country at day $Start$. Results of the parameter estimation and parameter identifiability analysis for both delay-based ad classic SEIR models are presented in Supplementary material 3, more details may be also found in corresponding Jupyter Notebook at https://gitlab.sirius-web.org/covid-19/dde-epidemiology-model.

According to the analysis for delay-based SEIR model: ${\beta }_{1}$ is identifiable parameter (i.e. if its value is changed from estimated ${\beta }_{1}$= 0.165 and fixed, then the model can not be successfully fitted by changing other parameters values); ${\beta }_{2}$—is non-identifiable in very small range [0, 0.0026] (i.e. its value may be anywhere in this range), value of ${\beta }_{3}$—is also non-identifiable in quite a small range [0, 0.029]. We can see that all three parameters can be identified quite precisely.

However, the parameter identifiability analysis has been demonstrated that for classic SEIR model: ${\beta }_{1}$ is not-identifiable in the range [0, 9.92], ${\beta }_{2}$, ${\beta }_{3}$ are not identifiable in the range [0, 10]. If we fix the value of one of these parameters anywhere in that range we may fit the model by changing values of other parameters. $Start$ is identifiable, where the identified value is day 83 (23.03.2020), while ${E}_{start}$ is non-identifiable in the range [10570, 2000] and $\alpha$ is non-identifiable in the range [0.57, 10]. Other parameters are also not-identifiable in large ranges.

Thus, the delay-differential modelling approach not only provides a smaller number of epidemiologically interpretable parameters, but also improves the identifiability of the model parameters compared to classic SEIR model.

### Initial model

As a basis for our model we used the previously created SEIR model^16^ of the COVID-19 epidemic. This model differs from the most SEIR models by differentiating between tested and non-tested infected subjects. It was created in the Systems Biology software COPASI^70^ which allows one to specify the kinetics of the process mechanistically. COPASI translates these specifications into differential equations which it integrates either as a function of time, or by requiring steady state. The software honors restrictions as specified in terms of algebraic equations and ‘events’ which instantaneously change numeric values of the model parameters triggered by logical expressions transiting from “false” to “true”. COPASI models are SBML compatible, and can be exported into the format. The latter greatly facilitates model reuse and reproduction.

### Data sources

Statistical data for Germany and France was taken from Our World In Data web site (https://ourworldindata.org/). This web-portal provides the data on the total number of cases, new cases each day, number of hospitalized, transferred to ICU patients, vaccinated individuals and total number of deaths.

**Figure 10:**
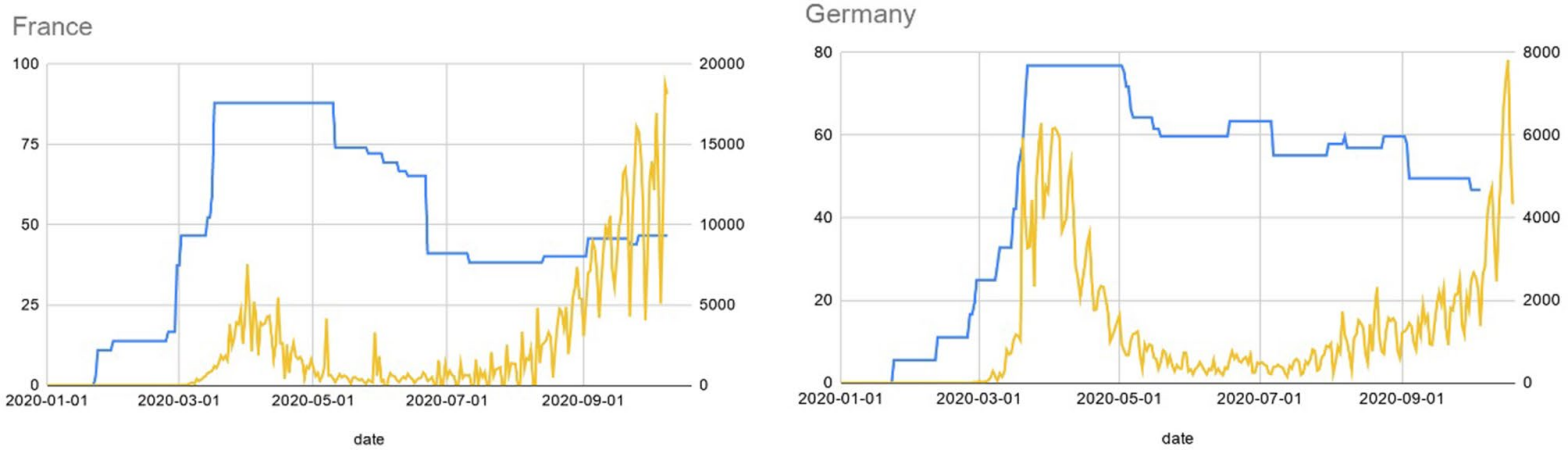
Stringency of government measures (blue) and new reported cases per day (yellow).

To take into account statistical data on government actions in the model we employed the Stringency Index developed by the Blavatnik School of Government of the University of Oxford^32^ which incorporates data on 10 different measures corresponded to: C1—School closing, C2—Workplace closing, C3—Public events cancellation, C4—Restriction on gatherings, C5—Public transport closing, C6—Stay at home requirements, C7—Internal movement, C8—International movement, H1—public information campaigns. Comparison between Stringency index and new registered cases is provided in Fig. 10.

### BioUML platform

BioUML (http://www.biouml.org) used in the study is an integrated Java platform for modeling of biological systems^71^. It supports different mathematical formulations for the model development including ODE, delay-based, algebraic systems, discrete events, agent-based, and stochastic modeling. The platform also incorporates a module for the automatic and manual parameter fitting to an experimental data. Models developed in the BioUML are based on main standards in systems biology: (1) SBML—Systems Biology markup Language^72^ for mathematical description and (2) SBGN for visual representation. A model can be built and edited in the platform as a visual diagram (e.g. in SBGN notation) based on which a Java code is generated for model simulations. Additionally, BioUML is integrated with Jupyter hub (https://jupyter.org/) for interactive data and model analysis as well as an essential and user-friendly tool for reproducibility of the simulation results.

### Parameter identifiability

Assessing of the parameter identifiability was conducted using a method implemented in the platform^73^. Identifiability analysis examines whether a set of model parameter values can be uniquely estimated from a given model and data set. According to the methodology, the agreement of experimental data with the observables predicted by the model is measured by an objective function, commonly the weighted sum of squared errors. The analysis goes as follows: one of the parameters is selected, its value is fixed and the model is fitted to observed data using other parameters. Then the fixed parameter value is changed in order to find a range of values for a given parameter in which the model could not be fitted to observed data. An area in which the model may be fitted regarding a given parameter value (inside this region) is the range in which this parameter is unidentifiable (its value cannot be uniquely found considering current data, model and set of parameters for estimation). Then the procedure repeats for the next model parameter.

### Supplementary Information

Supplementary Information 1.

Supplementary Information 2.

Supplementary Information 3.

## Data Availability

The data that support the findings of this study are available from https://ourworldindata.org/. Datasets selected for particular regions are also available through the web interface of BioUML software at https://gitlab.sirius-web.org/covid-19/dde-epidemiology-model. The developed models are available through the web interface of BioUML software at https://gitlab.sirius-web.org/covid-19/dde-epidemiology-model. Models are available both through visual representation in the platform and in Jupyter notebooks which allow users to reproduce simulation results and figures presented in this study.
