# Supplementary material for: Delay-differential SEIR modeling for improved modelling of infection dynamics
Source: Sci Rep. 2023 Aug 18;13:13439. doi: 10.1038/s41598-023-40008-9 (PMC10439236; doi:10.1038/s41598-023-40008-9)
Supplement: Supplementary file 1 — Supplementary Information 1. [file 41598_2023_40008_MOESM1_ESM.docx]

Delay-differential SEIR modeling for improved modelling of infection dynamics

Kiselev I.N.^1,2,3,*^, Akberdin I.R.^1,3,4^, Kolpakov F.A.^1,2,3^

^1^ BIOSOFT.RU, Ltd, Novosibirsk, Russia

^2^ FRC for Information and Computational Technologies, Novosibirsk, Russia

^3^ Sirius University, Sochi, Russia

^4^ Novosibirsk State University, Novosibirsk, Russia

*E-mail: axec@systemsbiology.ru

# Supplementary material 1: Model parameters

**Supplementary 1 Table S1.** Population parameters.

| **#** | **Symbol** | **Description** | **Germany** | **France** | **Source** |
| --- | --- | --- | --- | --- | --- |
| 1. | P | Initial population size | 8.37E7 | 6.7E7 | ourworldindata.org |
| 2. | $CF$ | Average number of contacts per day for one individual | 8.0 | 10.3 | [1] |
| 3. | $T_{Start}$ | Date when import of the infection started | 20.01.2020 | 15.01.2020 | [2] |
| 4. | $T_{Ended}$ | Date when import of the infection ended (borders were closed) | 16.03.2020 | | [3] |
| 5. | $I_{Started}$ | Infections imported per day at $T_{Started}$ | 0 cases | | Assumed |
| 6. | $I_{Ended}$ | Infections imported per day at $T_{Ended}$ | 500 cases | | Fitted |

**Supplementary 1 Table S2.** Processes fitted using formula (3 in the main text) and data from [4-5].

| **Process** | **Median [Quartiles]**  **(Observed)** | **Calculated quartiles** | **Results of the fitting** |
| --- | --- | --- | --- |
| Incubation period | 5.1 [2.2, 3.8, 5.1, 6.7, 11.5]  [4] | [2.13, 3.56, 5, 6.9, 11.6] | $0.14\cdot x(t-2)+0.04\cdot x(t-3)$ |
| Recovery with mild or no symptoms | 14 [8, 20]  [5] | 14.9 [8.13, 21.2] | $0.048\cdot x(t-3) + 0.0017\cdot x(t-4) +$  $+ 0.0166\cdot x(t-15)$ |
| Onset of severe symptoms | 5 [2 , 9]  [5] | 4.9 [2, 9] | $0.14 \cdot x(t) + 0.014\cdot x(t-5)$ |
| Release from hospital (without ICU) | 9 [6, 15]  [5] | 9.2 [5.8, 14.7] | $0.086\cdot x(t-3)+0.67\cdot x(t-14.9)$ |
|  |  |  |  |
|  | 13.8 [5.7, 27.7]  Fitted based on hospitalization data from ourworldindata.org | 13.8 [5.75, 27.7] | $0.058\cdot x(t)$ |
| Onset of critical symptoms | 1 [0 , 2]  [5] | 0.96 [0.4 , 1.9] | $0.717\cdot x(t)$ |
| Discharge from ICU | 20 [10 , 39 ]  [5] | 20.4 [9.6, 38.7] | $0.007\cdot x(t-3.583)+0.028\cdot x(t$-1.585) |
|  |  |  |  |

**Supplementary 1 Table S3.** Government interventions parameters.

| **#** | **Symbol** | **Description** | **Germany** | **France** | **Source** |
| --- | --- | --- | --- | --- | --- |
| 1. | $SI$ | Stringency index | Tabular data from source | | ourworldindata.org |
| 2. | $k_{V}$ | Individuals vaccinated per day either with first dose or with booster | Tabular data from source | | ourworldindata.org |
| 3. | $SI_{Delay}$ | Delay between government interventions enacting an their effect | 9 days | | Fitted |
| 4. | $SI_{Effect}$ | Stringency index efficacy | 0.74 | 0.94 | Fitted |
| 5. | $Q_{E^{T}}$ | Mobility limit for registered without symptoms | 0.3 | 0.17 | Fitted |
| 6. | $Q_{M}$ | Mobility limit for mildly symptomatic (not registered) | 0.2 | 0.45 | Fitted |
| 7. | $Q_{M^{T}}$ | Mobility limit for mildly symptomatic (registered) | 0.1 | 0.1 | Fitted |
| 8. | $Q_{H}$ | Mobility limit for severely symptomatic (not registered) | 0 (no mobility) | | Assumed |
| 9. | $Q_{H^{T}}$ | Mobility limit for severely symptomatic (registered) | 0 (no mobility) | | Assumed |

**Supplementary 1 Table S4.** Virus parameters (Wuhan strain, changes in consequent strains are listed in Table S6).

| **#** | **Symbol** | **Description** | **Germany** | **France** | **Source** |
| --- | --- | --- | --- | --- | --- |
| 1. | I | Infection probability upon contact (Wuhan variant) | 3.6% | | Fitted, corresponds to the range 0.7-16% [6-8] |
| 2. | $A_{F}$ | Fraction of asymptomatic among susceptible | 46% | | Fitted, corresponds to the range 20-75% [9] |
| 3. | $H_{F}$ | Fraction of severe symptoms (or worse) among symptomatic | 20% | 25% | Fitted, corresponds to the range 10-40%  [10-11] |
| 4. | $C_{F}$ | Fraction of critically ill among severe | 16% | 10% | Fitted |
| 5. | $D_{H}$ | Fatality rate for severe symptoms without ICU or without hospitalization. | 30% | 25% | Fitted |
| 6. | $D_{ICU}$ | Fatality rate for patients in ICU | 30% | | [12-13] |
| 7. | $T_{Immunity}$ | Immunity duration (both from vaccine and natural) | 180 days | | [14-15] |

**Supplementary 1 Table S5.** Parameters of testing for Covid-19 in the model.

| **#** | **Symbol** | **Description** | **Germany** | **France** | **Source** |
| --- | --- | --- | --- | --- | --- |
| 1. | $T_{E}$ | Percent of registered while in incubation period or asymptomatic. In brackets - starting date of new testing value. | 25%  (14.03.2020)  30%  (01.11.2020) | 16%  (14.03.2020) | Fitted |
| 2. | $T_{M}$ | Percent of registered while having mild symptoms. In brackets - starting date of new testing value. | 48%  (14.03.2020)  73%  (01.11.2020) | 50%  (14.03.2020)  60%  (28.09.2020) | Fitted |
|  |  |  |  |  |  |
| 3. | $T_{H}$ | Percent of registered with severe symptoms | 100% | | Assumed |

**Supplementary 1 Table S6.** New virus variants in the model.

| **Stain** | **Transmissibility** | **Infection coefficient (relative to Wuhan strain)** | **Germany** | | **France** | |
| --- | --- | --- | --- | --- | --- | --- |
|  |  |  | **Date of parameter change (fitted)** | $\boldsymbol{H}_{\boldsymbol{F}}$ | **Date of parameter change (fitted)** | $\boldsymbol{H}_{\boldsymbol{F}}$ |
| Wuhan Hu-1 | R0 = 2,5-3 [16-18] | 1 | 1  (01.01.2020) | 20% | 1  (01.01.2020) | 25% |
| B.1.177  (EU1) | R0 is in the same range as for the original strain [19] | 1 | 200  (20.07.2020) | 8% | 200 (20.07.2020) | 5% |
| B.1.1.7  (Alpha) | Is 43 to 90% more transmissible than the predecessor lineage [20] | 1.6 | 400  (03.02.2021) | 8% | 450  (25.03.2021) | 4% |
| B.1.617.2  (Delta) | R0=3.2-8 [21] | 2.3 | 518  (01.06.2021) | 3% | 550  (03.07.2021) | 2% |

REFERENCES

1. Del Fava, E., et al. The differential impact of physical distancing strategies on social contacts relevant for the spread of COVID-19. *BMJ Open* **11**(10), e050651 (2021).
2. Böhmer, M. M. et al. Investigation of a COVID-19 outbreak in Germany resulting from a single travel-associated primary case: a case series. *Lancet Infect. Dis.* **20**(8), 920-928 (2020).
3. European Commission. “COVID-19: Temporary Restriction on Non-Essential Travel to the EU.” *European Commission*. accessed 16 March 2020a. <https://eur-lex.europa.eu/legal-content/EN/TXT/HTML/?uri=CELEX:52020DC0115&from=EN>. (2020a).
4. Lauer, S. A., et al. The Incubation Period of Coronavirus Disease 2019 (COVID-19) From Publicly Reported Confirmed Cases: Estimation and Application. *Ann. Intern. Med*. **172**(9), 577-582 (2020).
5. Boëlle, P. Y., et al.. Trajectories of hospitalization in COVID-19 patients: an observational study in France. *J. Clin. Med.* **9**(10), 3148 (2020).
6. Cheng, H. Y., et al. Contact tracing assessment of COVID-19 transmission dynamics in Taiwan and risk at different exposure periods before and after symptom onset. *JAMA Intern. Med.* **180**(9), 1156-1163 (2020).
7. Phucharoen, C., Sangkaew, N., Stosic, K. The characteristics of COVID-19 transmission from case to high-risk contact, a statistical analysis from contact tracing data. *EClinicalMedicine* **27**, 100543 (2020).
8. Agrawal, A., & Bhardwaj, R. Probability of COVID-19 infection by cough of a normal person and a super-spreader. *Phys. Fluids* **33**(3), 031704 (2021).
9. Yanes-Lane, M., et al. Proportion of asymptomatic infection among COVID-19 positive persons and their transmission potential: A systematic review and meta-analysis. *PloS one* **15**(11), e0241536 (2020).
10. Wu, Z., McGoogan, J. M. Characteristics of and important lessons from the coronavirus disease 2019 (COVID-19) outbreak in China: summary of a report of 72 314 cases from the Chinese Center for Disease Control and Prevention. *JAMA* **323**(13), 1239-1242 (2020).
11. Garg, S., et al. Clinical trends among US Adults hospitalized with COVID-19, March to December 2020: a cross-sectional study. *Ann. Intern. Med.* **174**(10), 1409-1419 (2021).
12. Karagiannidis, C., Windisch, W., McAuley, D. F., Welte, T. and Busse, R. Major differences in ICU admissions during the first and second COVID-19 wave in Germany. *Lancet Respir. Med.* **9**(5), e47-e48, (2021).
13. Rimmelé, T., Pascal, L., Polazzi, S. and Duclos, A. Organizational aspects of care associated with mortality in critically ill COVID-19 patients. *Intensive Care Med.* **47**(1), 119-121 (2021).
14. Baraniuk, C. How long does covid-19 immunity last? *BMJ*, **373**, n1605 (2021).
15. Radbruch, A., Chang, H. D. A long-term perspective on immunity to COVID. *Nature* **595***,* 359-360 (2021).
16. Li, R., et al. Substantial undocumented infection facilitates the rapid dissemination of novel coronavirus (SARS-CoV-2). *Science* **368**(6490), 489-493 (2020).
17. Park, S. W., et al. Reconciling early-outbreak estimates of the basic reproductive number and its uncertainty: framework and applications to the novel coronavirus (SARS-CoV-2) outbreak. *J R Soc Interface* **17**(168), 20200144 (2020).
18. Xu, H., et al. Basic Reproduction Number of the 2019 Novel Coronavirus Disease in the Major Endemic Areas of China: A Latent Profile Analysis. *Public Health Front.*, **9,** 575315 (2021).
19. Hodcroft, E. B., et al. Spread of a SARS-CoV-2 variant through Europe in the summer of 2020. *Nature* **595**(7869), 707-712 (2021).
20. Davies, N. G. et al. Estimated transmissibility and impact of SARS-CoV-2 lineage B. 1.1.7 in England. *Science* **372**(6538), eabg3055 (2021).
21. Liu, Y., Rocklöv, J. The reproductive number of the Delta variant of SARS-CoV-2 is far higher compared to the ancestral SARS-CoV-2 virus. *J. Travel Med.* **28**(7), taab124 (2021).
