# Supplementary material for: Delay-differential SEIR modeling for improved modelling of infection dynamics
Source: Sci Rep. 2023 Aug 18;13:13439. doi: 10.1038/s41598-023-40008-9 (PMC10439236; doi:10.1038/s41598-023-40008-9)
Supplement: Supplementary file 2 — Supplementary Information 2. [file 41598_2023_40008_MOESM2_ESM.docx]

Delay-differential SEIR modeling for improved modelling of infection dynamics

Kiselev I.N.^1,2,3,*^, Akberdin I.R.^1,3,4^, Kolpakov F.A.^1,2,3^

^1^ BIOSOFT.RU, Ltd, Novosibirsk, Russia

^2^ FRC for Information and Computational Technologies, Novosibirsk, Russia

^3^ Sirius University, Sochi, Russia

^4^ Novosibirsk State University, Novosibirsk, Russia

*E-mail: axec@systemsbiology.ru

## Supplementary material 2: Automatic generation models for other countries

Here we present results of automatic model generation for several European and non-European countries. As a basis we took a model fitted for Germany data and used it for automatic fitting based on statistical data for other countries. Of course, the resulting models are not final products, but they may be used as a starting point for full-fledged models describing Covid-19 pandemic in correspondent countries. Additional information may be found at

<https://gitlab.sirius-web.org/covid-19/dde-epidemiology-model>.

**Supplementary 2 Table S1**. Parameters which values are estimated for different countries.

| **Name** | **Description** | **Initial guess** | **Lower boundary** | **Upper boundary** |
| --- | --- | --- | --- | --- |
| $SI_{Effect}$ | Stringency index efficacy | 0.74 | 0.3 | 1 |
| $CF$ | Average number of contacts per day for one individual | 8 | 1.6 | 100 |
| $Q_{E^{T}}$ | Mobility limit for registered without symptoms | 0.3 | 0 | 1 |
| $Q_{M}$ | Mobility limit for mildly symptomatic (not registered) | 0.2 | 0 | 1 |
| $Q_{M^{T}}$ | Mobility limit for mildly symptomatic (registered) | 0.1 | 0 | 1 |
| $T_{E}$ | Percent of registered while in incubation period or asymptomatic. In brackets - starting date of new testing value. | 25% | 0% | 100% |
| $T_{M}$ | Percent of registered while having mild symptoms. In brackets - starting date of new testing value. | 48% | 0% | 100% |
| $I_{Ended}$ | Infections imported per day at $T_{Ended}$ | 500 cases | 0 cases | 3000 cases |

**Supplementary 2 Table S2**. Parameter values estimated for individual countries. MSD - mean squared deviation.

| **Country** | $CF$ | $\boldsymbol{S}\boldsymbol{I}_{\boldsymbol{Effect}}$ | $\boldsymbol{I}_{\boldsymbol{Ended}}$ | $\boldsymbol{Q}_{\boldsymbol{M}}$ | $\boldsymbol{T}_{\boldsymbol{E}}$ | $\boldsymbol{T}_{\boldsymbol{M}}$ | $\boldsymbol{Q}_{\boldsymbol{E}^{\boldsymbol{T}}}$ | $\boldsymbol{Q}_{\boldsymbol{M}^{\boldsymbol{T}}}$ | MSD |
| --- | --- | --- | --- | --- | --- | --- | --- | --- | --- |
| **Brazil** | 4 | 0.55 | 2485 | 0.3 | 0.6 | 0.54 | 0.99 | 0.41 | 7308271 |
| **Argentina** | 4.18 | 0.32 | 1471 | 0.15 | 0.57 | 0.12 | 0.82 | 0.19 | 1561563 |
| **Russia** | 3.32 | 0.32 | 1936 | 0.05 | 0.49 | 0.25 | 0.83 | 0.58 | 142823 |
| **Italy** | 9.5 | 0.99 | 5112 | 0.26 | 0.17 | 0.53 | 0.86 | 0.94 | 134092 |
| **Spain** | 5.15 | 0.61 | 198 | 0.07 | 0.53 | 0.58 | 0.96 | 0.08 | 115627 |
| **Poland** | 7.81 | 0.77 | 1286 | 0.19 | 0.06 | 0.48 | 0.19 | 0.06 | 81643 |
| **Mexico** | 6.49 | 0.40 | 1776 | 0.04 | 0.44 | 0.04 | 0.41 | 0.06 | 737347 |
| **Czechia** | 6.09 | 0.72 | 183 | 0.1 | 0.56 | 0.36 | 0.20 | 0.76 | 725553 |
| **Portugal** | 6.02 | 0.60 | 1102 | 0.28 | 0.11 | 0.11 | 0.31 | 0.24 | 22143 |
| **Austria** | 8.47 | 0.86 | 251 | 0.04 | 0.11 | 0.22 | 0.43 | 0.21 | 204447 |
| **Sweden** | 6.57 | 0.81 | 287 | 0.22 | 0.04 | 0.21 | 0.89 | 0.35 | 18745 |
| **Japan** | 4.19 | 0.54 | 226 | 0.85 | 0.19 | 0.36 | 0.43 | 0.59 | 134672 |
| **Greece** | 8.62 | 0.97 | 242 | 0.23 | 0.06 | 0.54 | 0.52 | 0.51 | 6534 |
| **South Korea** | 4.31 | 0.43 | 507 | 0.03 | 0.06 | 0.08 | 0.46 | 0.95 | 5362 |


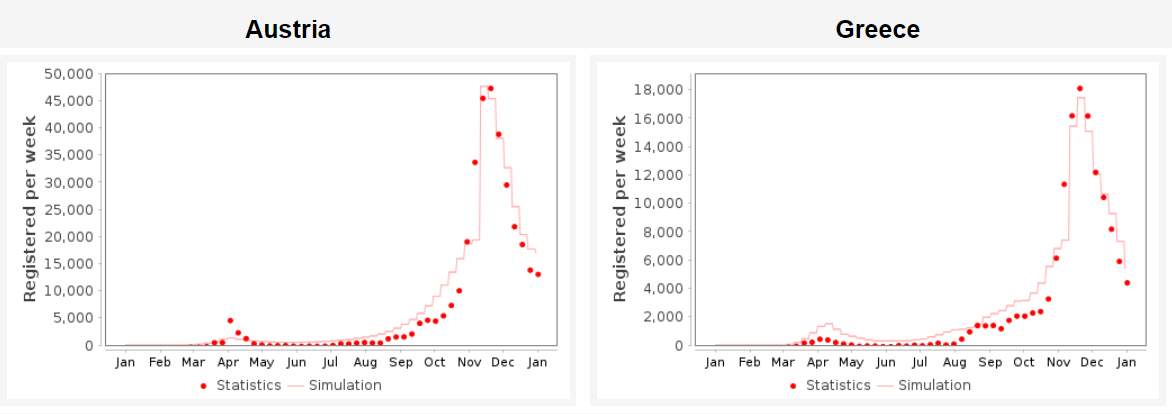

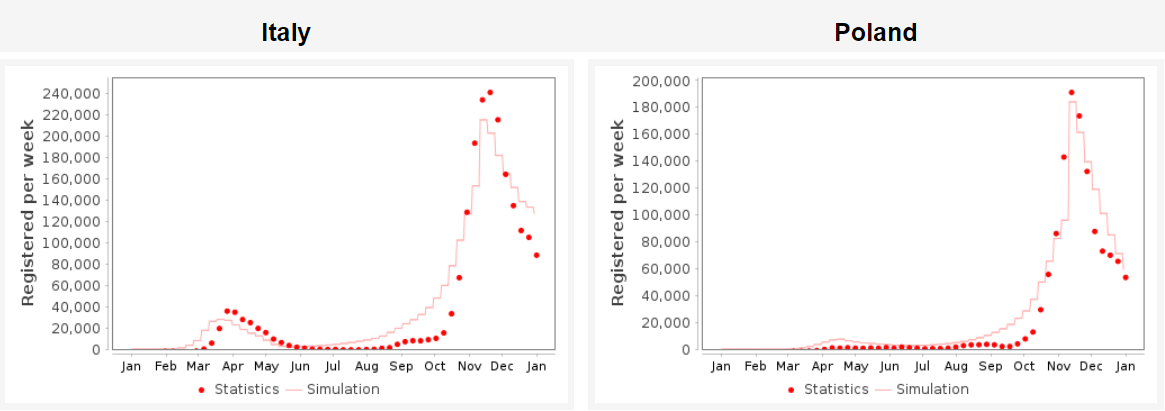


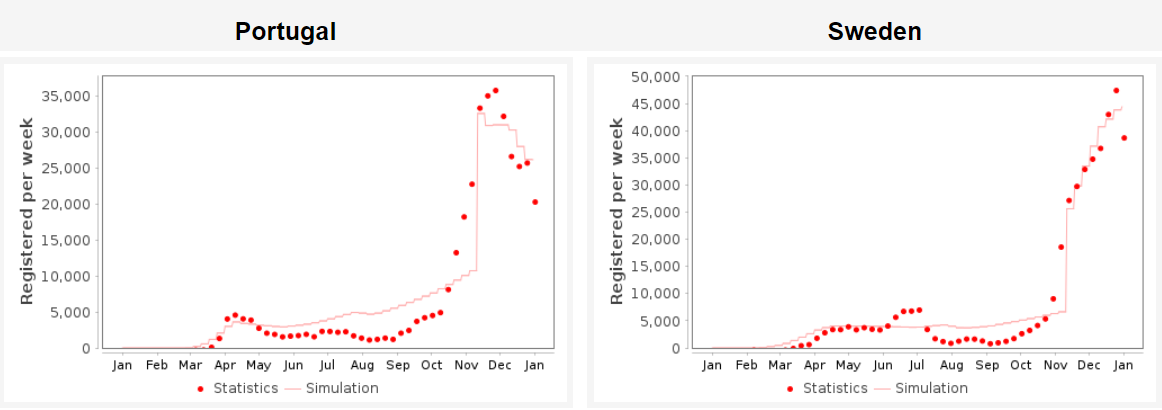


**Supplementary 2 Fig. S1.** Simulation results for autogenerated models for 6 European countries. Axis Y - cases per week. The curve - simulation results, while dots -statistics data.


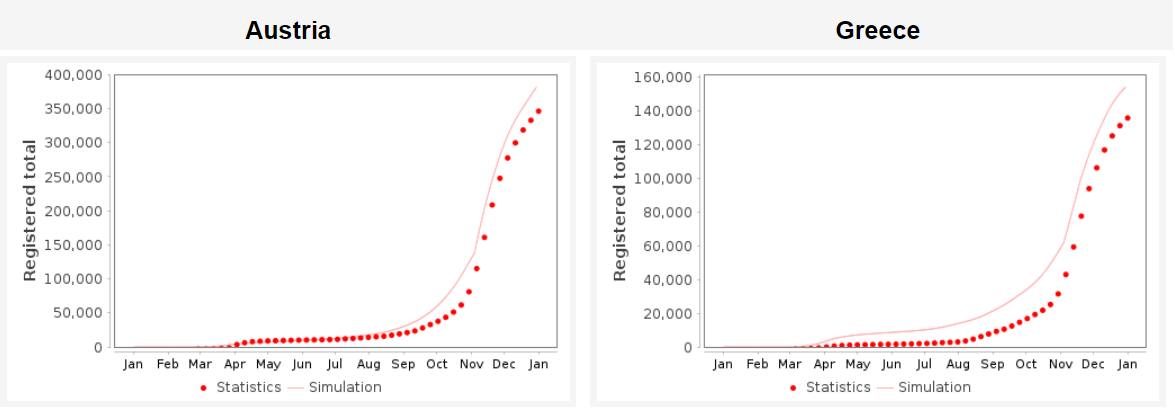


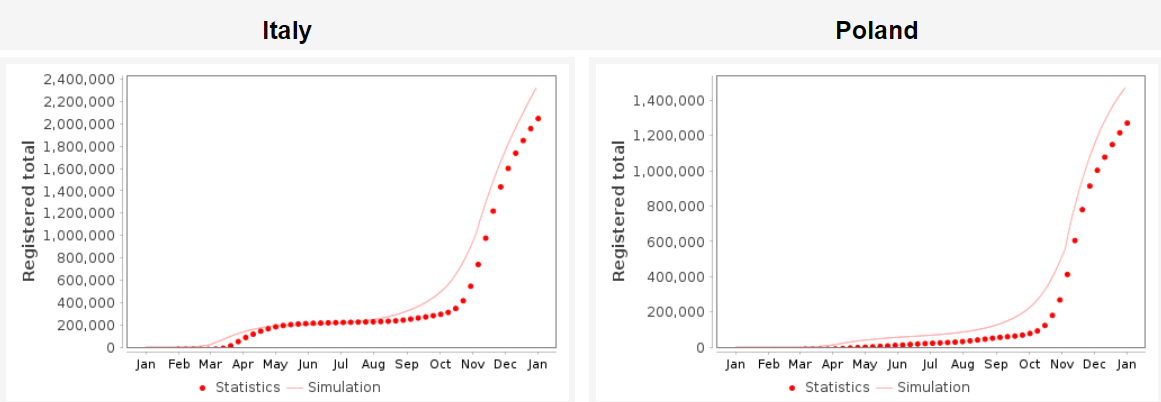


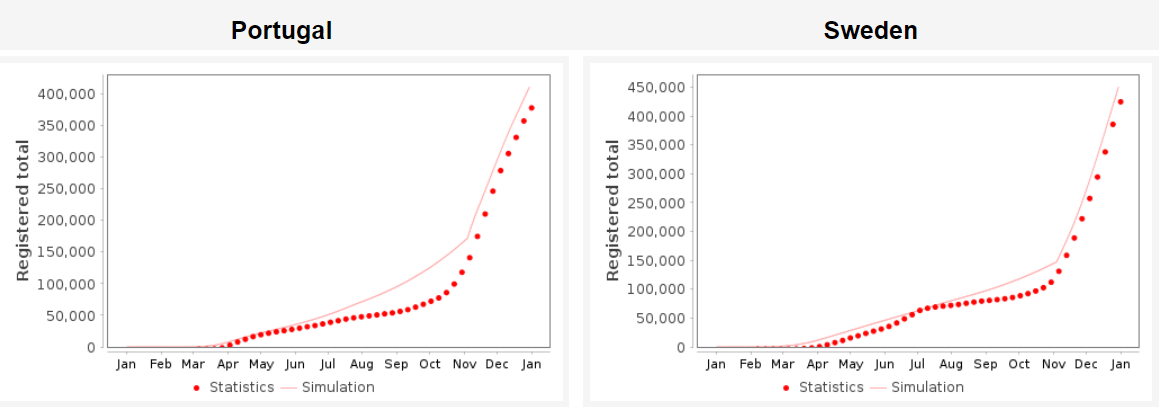


**Supplementary 2 Fig. S2.** Simulation results for autogenerated models for 6 European countries. Axis Y - total number of cases. The curve - simulation results, while dots -statistics data.


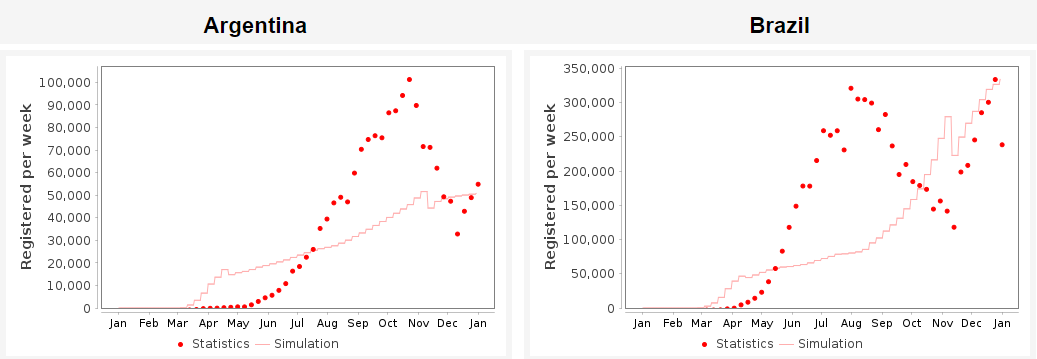


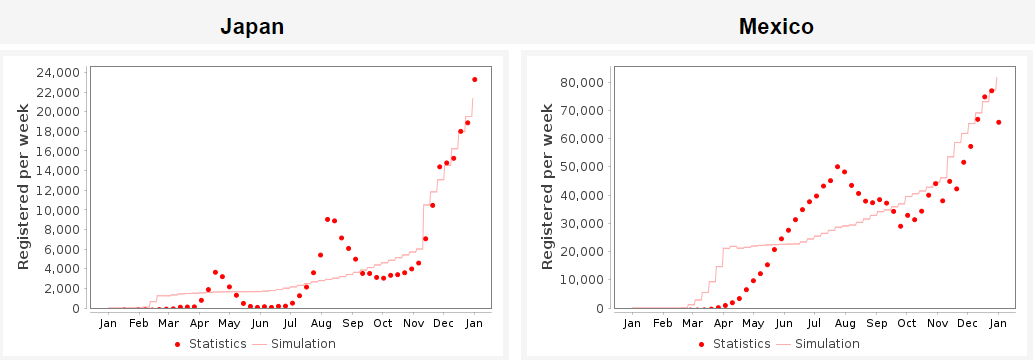


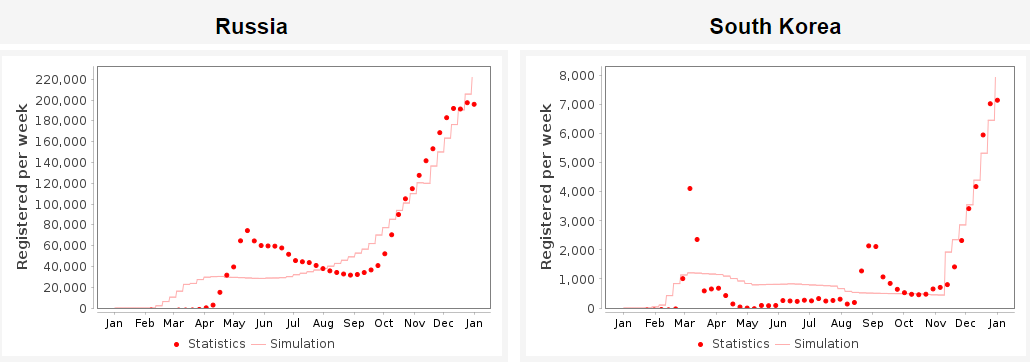


**Supplementary 2 Fig. S3.** Simulation results for autogenerated models for 6 non-European countries. Axis Y - cases per week. The curve - simulation results, while dots -statistics data.


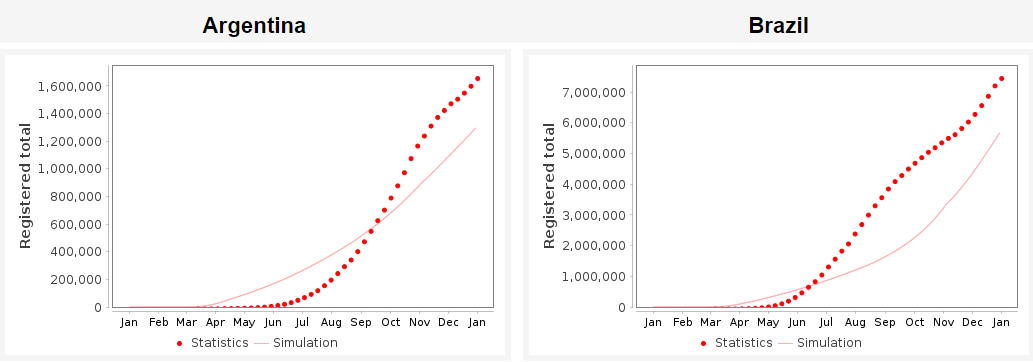


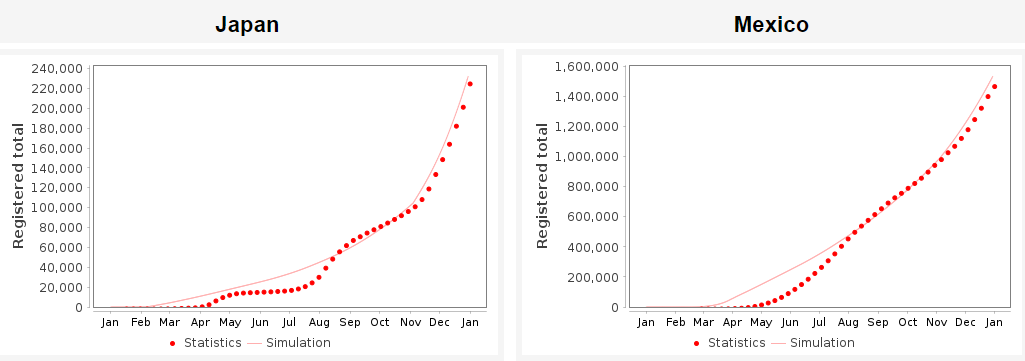


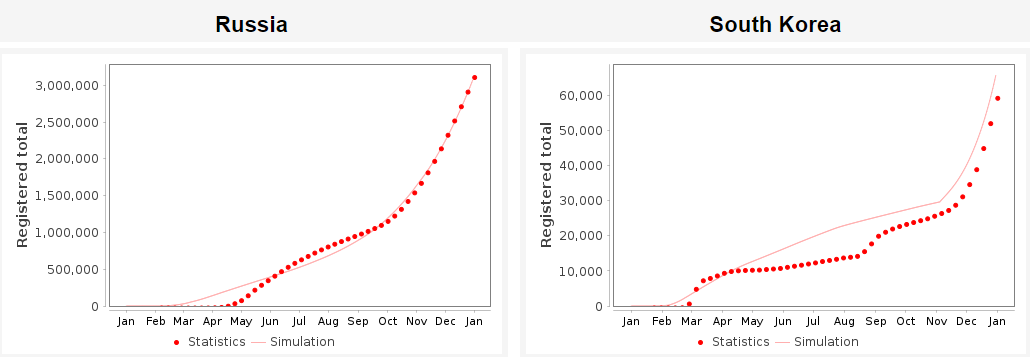


**Supplementary 2 Fig. S4.** Simulation results for autogenerated models for 6 non-European countries. Axis Y - total number of cases. The curve - simulation results, while dots -statistics data.
