# Supplementary material for: Delay-differential SEIR modeling for improved modelling of infection dynamics
Source: Sci Rep. 2023 Aug 18;13:13439. doi: 10.1038/s41598-023-40008-9 (PMC10439236; doi:10.1038/s41598-023-40008-9)
Supplement: Supplementary file 3 — Supplementary Information 3. [file 41598_2023_40008_MOESM3_ESM.docx]

Delay-differential SEIR modeling for improved modelling of infection dynamics

Kiselev I.N.^1,2,3,*^, Akberdin I.R.^1,3,4^, Kolpakov F.A.^1,2,3^

^1^ BIOSOFT.RU, Ltd, Novosibirsk, Russia

^2^ FRC for Information and Computational Technologies, Novosibirsk, Russia

^3^ Sirius University, Sochi, Russia

^4^ Novosibirsk State University, Novosibirsk, Russia

*E-mail: axec@systemsbiology.ru

# Supplementary material 3: Parameters Identifiability

Here we present results of parameter identifiability analysis for delay-differential model and SEIR classic model. Both models were fitted to statistical data on daily new cases and cumulative deaths during Covid-19 pandemics in Germany in the first 180 days of 2020. Then, the identifiability analysis was performed to identify non-identifiability ranges for model parameters. Non-identifiability range is the range for selected parameter value in which the model still can be fitted using other parameters (while selected parameter is fixed). Ideally, an identifiable parameter is a parameter with the range corresponding to one point, i.e. if the parameter value is changed in any way, model can not be fitted anymore. The analysis starts from initially fitted values and makes steps in both directions, increasing/decreasing the value of selected parameters and checking if the model can still be fitted. Of course, it can not always be automatically detected if the model's fitting is not acceptable. Red line on all charts is a threshold for distance between simulated values and statistical data. The analysis stops if the threshold is exceeded by objective function. However, not all values below the red line are acceptable and manual selection and check is required to determine the non-identifiability range. More detailed charts with simulation results for different parameter values are given at <https://gitlab.sirius-web.org/covid-19/dde-epidemiology-model>

**Supplementary 3 Table S1.** Classic SEIR model’s parameters. The smaller range of non-identifiability is the more identifiable the parameter is.

| Symbol | Description | Estimated value | Estimation boundaries | Non-identifiability range |
| --- | --- | --- | --- | --- |
| $\beta_{1}$ | Infection rate of exposed individuals | 0.165 | [0,10] | [0, 9.92] |
| $\beta_{2}$ | Infection rate for mildly ill | 2.6E-10 | [0,10] | [0, 10] |
| $\beta_{3}$ | Infection rate for severely ill | 4.5E-9 | [0,10] | [0, 10] |
| $\alpha$ | Rate of symptoms appear | 0.64 | [0,10] | [0.57, 10] |
| $\delta$ | Rate of symptoms worsening | 1 | [0,10] | [5.6, 10] |
| $\mu$ | Rate of dying | 3.11 | [0,10] | [3.1, 10] |
| $\gamma_{1}$ | Rate of recovery mildly ill | 8,5 | [0,10] | [0, 10] |
| $\gamma_{2}$ | Rate of recovery for severely ill | 4.5 | [0,10] | [0, 10] |
| $Start$ | Time of infection import | 83 | [0,120] | [83] |
| $E_{Start}$ | Number of infected individuals imported | 10570 | [0,20000] | [10570, 20000] |

**Supplementary 3 Table S2.** Delay-based model’s parameters. The smaller range of non-identifiability is the more identifiable the parameter is.

| Symbol | Description | Estimated value | Boundaries | Non-identifiability range |
| --- | --- | --- | --- | --- |
| $\beta_{1}$ | Infection rate of exposed individuals | 0.165 | [0,10] | [ 0.165] |
| $\beta_{2}$ | Infection rate of mildly ill | 2.6E-10 | [0,10] | [0, 0.0026] |
| $\beta_{3}$ | Infection rate of severely ill | 4.5E-9 | [0,10] | [0, 0.029] |
| $Start$ | Time of infection import | 78 | [0,120] | [78] |
| $E_{Start}$ | Number of infected individuals imported | 20000 | [0,20000] | [17000 , 2000] |


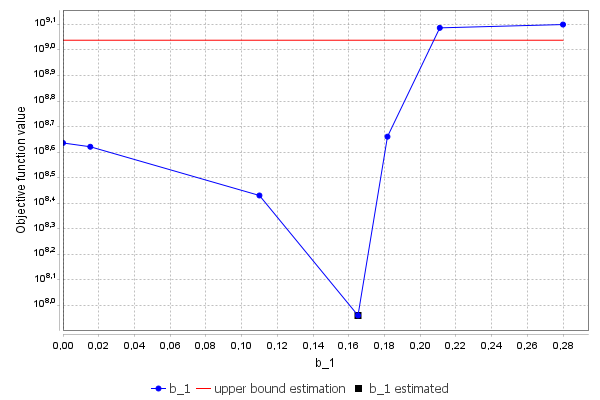


**Supplementary 3 Fig. S1**. Identifiability of the parameter $\beta_{1}$ in the delay-based model. Parameter is identifiable, identified value is 0.165.


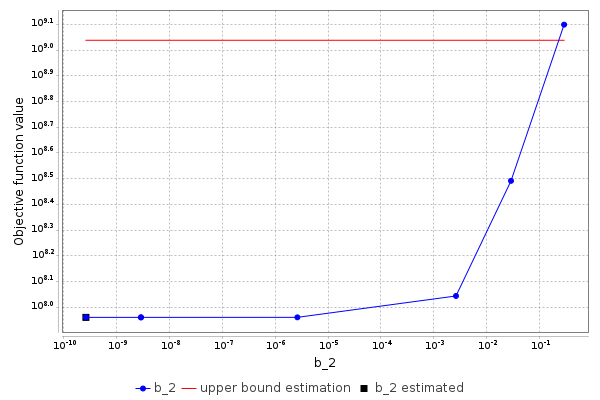


**Supplementary 3 Fig. S2**. Identifiability of the parameter $\beta_{2}$ in the delay-based model. Parameter is non identifiable in a very small range [0, 0.0026].


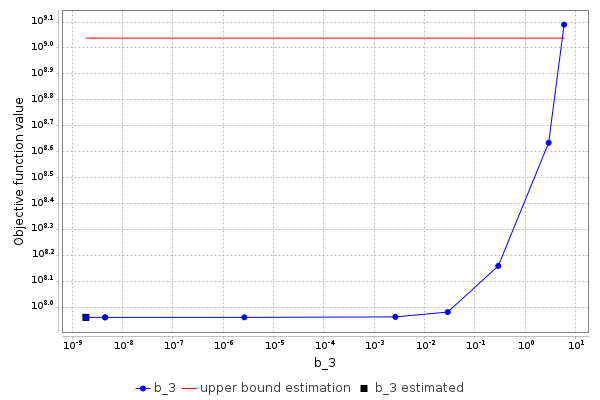


**Supplementary 3 Fig. S3.** Identifiability of the parameter $\beta_{3}$ in the delay-based model. Parameter is non identifiable in small range [0, 0.029].


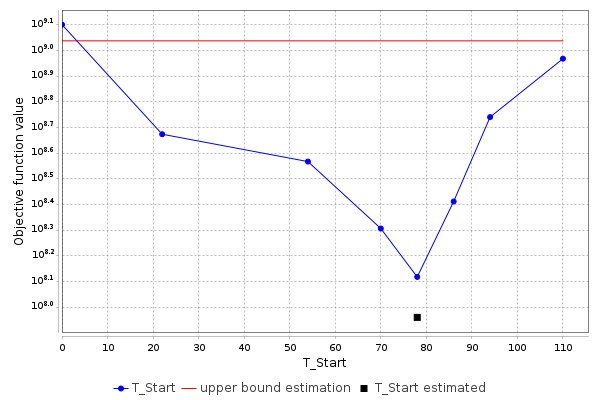


**Supplementary 3 Fig. S4.** Identifiability of the parameter $Start$ (day of infection import) in the delay-based model. Note that black square (initial estimation of the parameter) is slightly lower than the blue circle. It means that during parameter identifiability, a slightly worse solution was found with fixed Start = 78. Still the parameter is quite well identifiable.


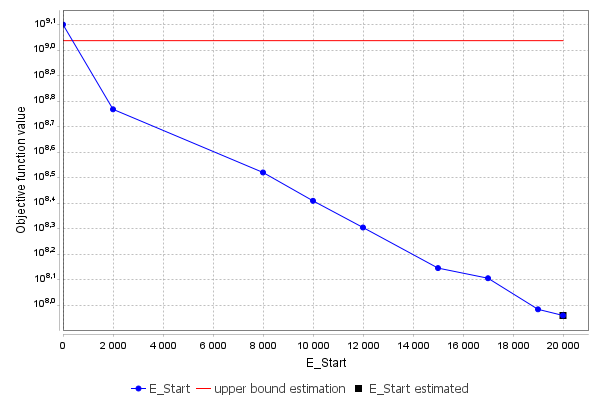


**Supplementary 3 Fig. S5.** Identifiability of the parameter $E_{Start}$ (number of imported cases at day Start) in the delay-based model.


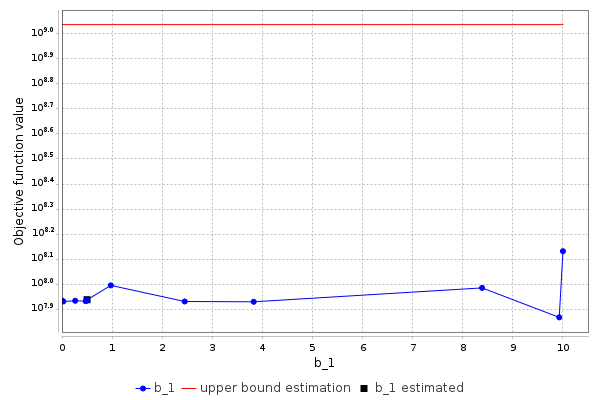


**Supplementary 3 Fig. S6.** Identifiability of the parameter $\beta_{1}$ in the classic SEIR model. Parameter is non-identifiable in range [0, 9.92].


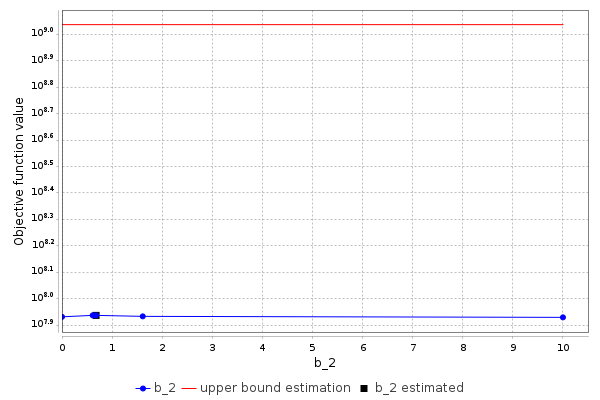


**Supplementary 3 Fig. S7.** Identifiability of the parameter $\beta_{2}$ in the classic SEIR model. Parameter is non-identifiable in range [0, 10].


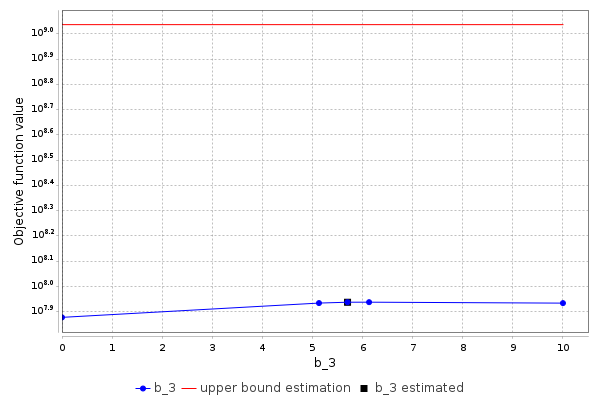


**Supplementary 3 Fig. S8.** Identifiability of the parameter $\beta_{3}$ in the classic SEIR model. Parameter is non-identifiable in range [0, 10].


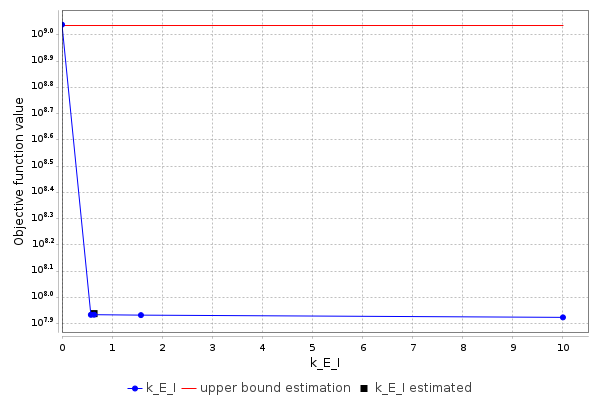


**Supplementary 3 Fig. S9.** Identifiability of the parameter $\alpha$ in the classic SEIR model. Parameter is non-identifiable in range [0.57, 10].


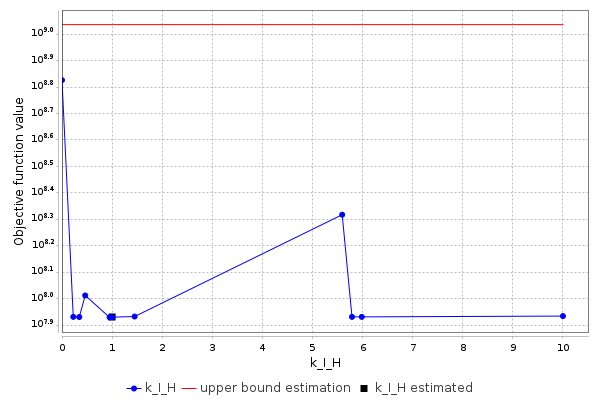


**Supplementary 3 Fig. S10** Identifiability of the parameter $\delta$ in the classic SEIR model. Parameter is non-identifiable in range [5.6, 10].


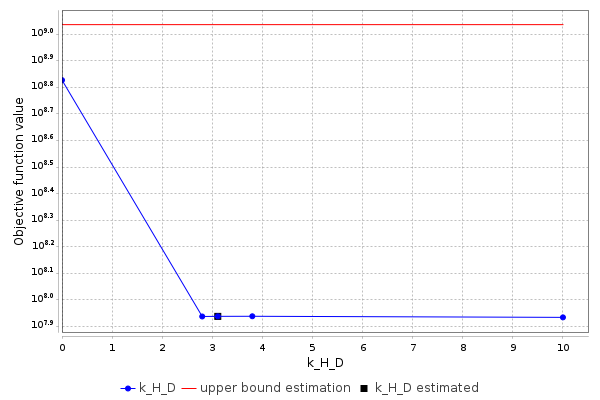


**Supplementary 3 Fig. S11.** Identifiability of the parameter $\mu$ in the classic SEIR model.Parameter is non-identifiable in range [3.1,10].


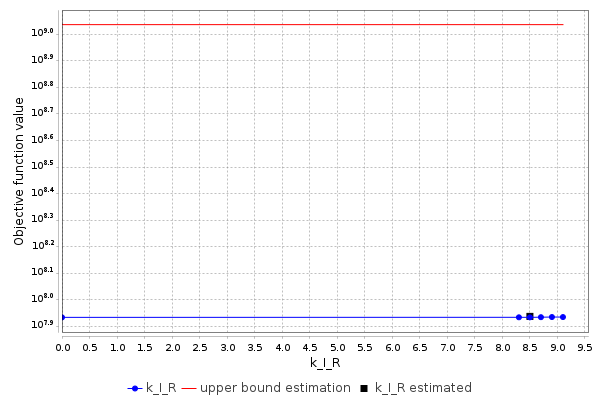


**Supplementary 3 Fig. S12.** Identifiability of the parameter $\gamma_{1}$ in the classic SEIR model. Parameter is non-identifiable in range [0,10].


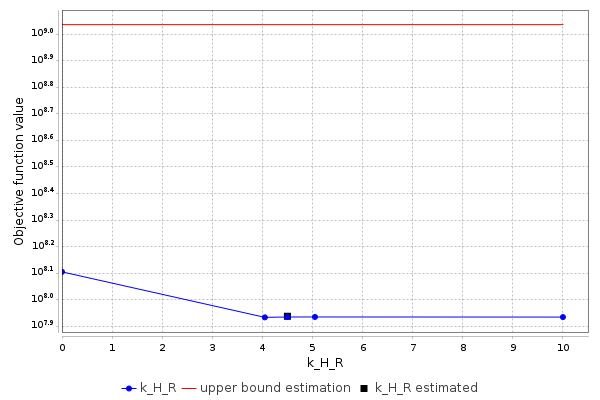


**Supplementary 3 Fig. S13.** Identifiability of the parameter $\gamma_{2}$ in the classic SEIR model. Parameter is non-identifiable in range [0,10].


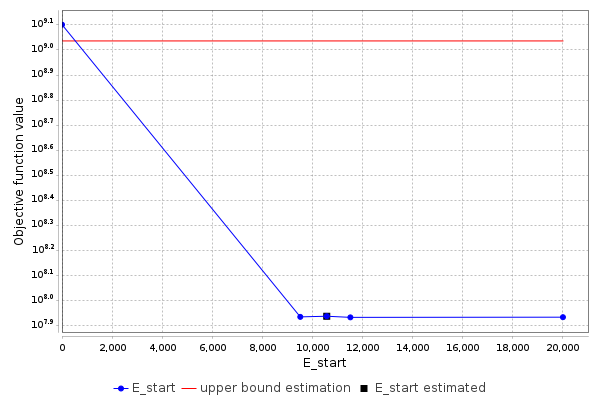


**Supplementary 3 Fig. S14.** Identifiability of the parameter $E_{Start}$ (number of infected individuals imported) in the classic SEIR model. Parameter is partially identifiable in range [10580,20000].


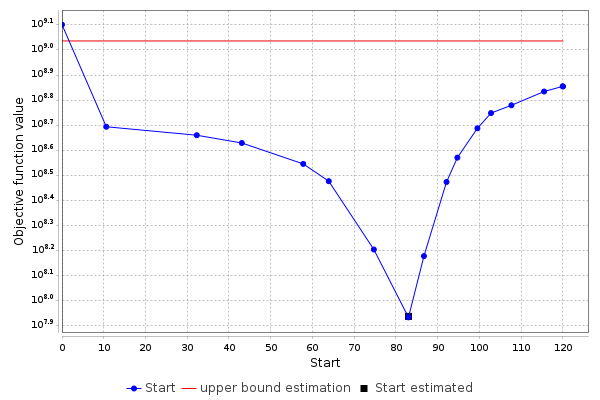


**Supplementary 3 Fig. S15.** Identifiability of the parameter $Start$ (time when infection was imported) in the classic SEIR model. Parameter is identifiable, identified value: day 83 (23.03.2020).
